# Downregulation of FTO aggravates osteoarthritis with obesity by erasing m^6^A methylation of PDP2

**DOI:** 10.1016/j.mmr.2026.100054

**Published:** 2026-07-17

**Authors:** Xu-Ying Sun, Jing-Yue Su, Xin Chen, Yi-Xin Yang, Sheng-Wu Yang, Chun-Wu Zhang, Yu-Sheng Li, Jian Xu, Zhen-Han Deng

**Affiliations:** a Department of Orthopedics, Tongji Hospital, Tongji Medical College, Huazhong University of Science and Technology, Wuhan 430030, China; b Department of Orthopedics, the First Affiliated Hospital of Wenzhou Medical University, Wenzhou 325000, Zhejiang, China; c Geriatrics Center, the First Affiliated Hospital of Wenzhou Medical University, Wenzhou 325000, Zhejiang, China; d Department of Epidemiology, University of California, Los Angeles, CA 90095, USA; e Department of Orthopedics, Xiangya Hospital, Central South University, Changsha 410008, China; f Department of Orthopedics, the First Affiliated Hospital, Zhejiang University School of Medicine, Hangzhou 310003, China

**Keywords:** Osteoarthritis (OA), Lipid deposition, Fat mass and obesity associated gene (FTO), Pyruvate dehydrogenase phosphatases 2 (PDP2), YTH N^6^-methyladenosine RNA binding protein 2 (YTHDF2)

## Abstract

**Background:**

Osteoarthritis (OA) is currently the most common age-related degenerative joint disease, but there remains a lack of disease-modifying therapy for OA treatment. This study aimed to elucidate the critical roles and underlying mechanism of the fat mass and obesity associated gene (FTO) in OA pathogenesis induced by high fat diet (HFD) and HFD coupled with destabilized medial meniscus (DMM) surgery, and to ascertain the synergistic action of the two in OA pathogenesis.

**Methods:**

Mouse models were established using solely HFD feeding and combined HFD feeding and DMM surgery. Primary mouse chondrocytes were treated with palmitic acid (PA) and interleukin-1β (IL-1β) to simulate lipotoxicity and inflammation. FTO expression was modulated via genetic, adenoviral, or pharmacological methods. Downstream pyruvate dehydrogenase phosphatases 2 (PDP2) and YTH N^6^-methyladenosine RNA binding protein 2 (YTHDF2) were similarly manipulated by siRNA or overexpression plasmids; adenovirus-mediated knockdown/overexpression of *Pdp2* was applied *in vivo*. Mechanistic studies included RNA sequencing (RNA-Seq) and methylated RNA immunoprecipitation sequencing (MeRIP-Seq).

**Results:**

The results revealed reduced FTO expression in the cartilage of both obese OA patients and mouse models. Genetically, adenovirus-induced or pharmacologically-induced FTO inhibition exacerbated OA progression in HFD-fed and HFD+DMM mice. Mechanistically, *Fto* knockdown downregulated the PDP2 level in an m^6^A-dependent manner via YTHDF2. *Pdp2* knockdown exacerbated OA progression in HFD-fed and HFD+DMM mice, whereas PDP2 overexpression markedly alleviated cartilage degeneration. Moreover, YTHDF2 overexpression reversed the role of *Fto* knockdown in lipid deposition and cartilage degeneration.

**Conclusions:**

Our study identifies that downregulation of FTO exerts a pivotal effect on OA with obesity. FTO drives disease progression by regulating PDP2 activity, and YTHDF2 mediates the m^6^A modification of FTO to PDP2. Targeting the FTO/YTHDF2/PDP2 axis offers promising therapeutic potential for OA treatment.

## 1 Background

Osteoarthritis (OA), the most prevalent age-related joint disease in elderly people [1], is characterized by progressively developing joint disability and pain, and currently affects approximately 250 million individuals worldwide [2], [3]. Unfortunately, there is still a lack of disease-modifying therapy for this disease. OA is known to impact multiple tissue structures within the joint and mainly manifests as articular cartilage destruction, including chondrocyte degeneration, apoptosis, extracellular matrix degradation, as well as pathological changes in the subchondral bone, synovial membrane, ligament, joint capsule, and other structures [4]. Numerous biochemical and biomechanical factors (e.g., release of inflammatory factors, abnormal mechanical stress) are believed to play important roles in disrupting the metabolic homeostasis of OA cartilage [5], [6], [7]. However, to date, the specific pathological mechanism of OA has not been fully elucidated. It is generally deemed that OA occurrence is associated with a range of factors including aging, obesity, inflammation, trauma, as well as genetic factors [8]. At present, the established therapeutic strategies for OA mainly involve symptom-controlling drugs (e.g., acetaminophen, non-steroidal anti-inflammatory drugs, hyaluronic acid), disease-modifying drugs, and cartilage protective agents (e.g., glucosamine, diacetoreine, chondroitin sulfate), but the treatment efficiency and application prospects remain unsatisfactory [9]. Late-stage treatment of OA often requires arthroplasty, whose high medical costs and potential complications impose huge psychological stress and economic burden on the patients and society. Therefore, elucidating the pathogenesis of OA, identifying effective targets, and implementing early interventions to delay or block OA progression serve primary goals for disease management.

Obesity is one of the main risk factors leading to OA. In addition to the stress damage that overweight imposes on articular cartilage, chronic inflammation of the cartilage and its surrounding tissues, which is induced by lipid deposition, is considered the initial driver of cartilage degeneration [10], [11], [12]. Under normal circumstances, chondrocytes are embedded in the extracellular matrix, where they maintain cartilage homeostasis and activate anabolic pathways to regulate the production and outcome of cartilage matrix. Upon the occurrence of OA, chondrocytes tend to produce more matrix metalloproteinases (MMPs), which accelerate the degradation of various cartilage matrix components, such as cartilage aggregating proteoglycan (aggrecan) and type II collagen (collagen II). Simultaneously, the master transcription factor sex determining region Y-box 9 (SOX9), which regulates multiple events in the process of cartilage regeneration [13], [14], is also impaired. Recent studies have revealed that OA patients exhibit significantly increased lipid deposition in the articular cartilage, which can even predate the onset of obvious joint lesions [15], [16]. Peroxisome proliferator-activated receptor (PPAR) signaling, an important pathway in lipid metabolism, is also identified to be involved in the occurrence and development of OA [15], [17]. However, the roles of lipid deposition in cartilage metabolism and OA pathogenesis have not been fully elucidated, with key molecules involved requiring further exploration. Among free fatty acids (FAs), palmitic acid (PA) is the most important component in saturated FAs and is commonly used to establish high-fat models *in vitro* [18], [19]. Interleukin-1β (IL-1β) is widely employed to induce OA and exacerbate inflammation in chondrocytes. In our study, co-simulation with PA and IL-1β was applied to investigate the combined role of inflammation and high fat in chondrocytes.

N^6^-methyladenine (m^6^A) modification represents the most abundant chemical modification on mRNA, affecting almost all aspects of RNA metabolism, including splicing, translation, and mRNA stability [20]. A growing number of studies have shown that m^6^A is involved in the occurrence and development of various diseases through three core steps: “writing”, “reading”, and “erasure”. Specifically, m^6^A modification is mediated by a methyltransferase complex known as “writer” [e.g., methyltransferase-like (METTL) 3, METTL14, Wilms tumor 1-associated protein (WTAP), Vir like m^6^A methyltransferase associated protein (VIRMA), zinc-finger CCCH-type containing 13 (ZC3H13), RNA-binding motif protein 15 (RBM15)], and is removed by demethylase, namely “eraser” [fat mass and obesity associated gene (FTO) and AlkB homolog 5 (ALKBH5)] [21], [22]. Among them, FTO is the most affected “writer” by obesity. It’s a newly discovered obesity-related gene and a pathogenic gene associated with obesity-induced OA [23], [24]. FTO-mediated m^6^A demethylation of pre-miR-3591 alleviates OA [25]. Additionally, FTO demethylates suppressor of variegation, enhancer of zeste, trithorax, and myeloid nervy DEAF-1 domain-containing protein 2 (SMAD2), protecting cartilage and indicating its role in mitigating inflammation-induced OA [26]. However, its involvement in regulating the process of lipid deposition and degeneration in chondrocytes, as well as the underlying mechanism, remains poorly understood. Despite these advances, the role of FTO in obesity-associated OA, particularly in chondrocyte lipid metabolism and degeneration, remains poorly understood. Moreover, it is unknown whether FTO regulates its downstream targets through m^6^A -dependent mechanisms in this context. Here, we aim to identify the direct downstream effector of FTO and to identify whether targeting this axis may represent a promising therapeutic strategy in OA.

## 2 Materials and methods

### 2.1 Patient samples

OA cartilage was collected from OA patients who underwent knee arthroplasty in Tongji Hospital in Wuhan. The information of the patients was listed in **Additional file 1:** Table S1. The collection of human cartilage was approved by the Ethics Committee of Tongji Hospital, Tongji Medical College, Huazhong University of Science and Technology (TJ-IRB202503074). Thirty-three cartilage samples were divided into body mass index (BMI) <27 or ≥27 kg/m^2^.

### 2.2 Animal model

Wild-type mice were purchased from the Experimental Animal Center of Tongji Hospital, Huazhong University of Science and Technology. All the experiments were approved by the Ethics Committee on Animal Experimentation of the Tongji Hospital (TJH-202209026). One hundred and twenty 8-week-old male mice were fed with high fat diet (HFD, rodent diet with 60% kcal fat, M10160, Moldiets, Shanghai, China) or normal chow diet (NCD, rodent maintenance diet with 9.4% kcal fat, 1016706714625204224, KeaoXieli Feed Co., Ltd., Beijing, China) for 4 consecutive weeks, then the mice were subject to sham or destabilized medial meniscus (DMM) surgery at 12 weeks of age as previously described (*n*=6−10 each group) [27] and continued with HFD for another 8 weeks, mice were sacrificed at 20-week-old. Blood glucose levels were measured using a glucometer with glucose testing strips after the mice were fasted for 12 h overnight every two weeks; body weight was measured every two weeks for each group.

For adenovirus (Ad) injection, Ad-shCtrl, Ad-shFTO (target sequence CGATACAAACTTTGCACCGAT), and Ad-shPDP2 (pyruvate dehydrogenase phosphatases 2, target sequence CGGAGTACCAAATTCAGTGTT) articular injection, Ad-shFTO, Ad-shPDP2, and relative scrambled shCtrl RNAi were designed and packaged by Genechem Co., Ltd. (Shanghai, China). Eight-week-old wild-type male mice were fed with HFD for 4 consecutive weeks, then the mice were subject to sham or DMM surgery at 12 weeks of age, 10 µl of [1×10^9^ plaque-forming units (PFUs)] were injected into the right knee joint cavity 1 week after DMM or sham surgery using a 33 G needle (Hamilton, Bonaduz, GR, Switzerland) once a week for 7 weeks (*n*=6−8 each group).

For FB23 (FTO inhibitor) injection, 8-week-old wild-type male mice were fed with HFD for 4 consecutive weeks, then the mice were subject to sham or DMM surgery at 12 weeks of age and continued with HFD for another 8 weeks, 3 mg/kg FB23 was injected into the knee joint cavity once a week for 7 weeks after surgery (*n*=7−8 each group), mice were sacrificed at 20 weeks old.

Conditional cartilage Col2a1-CreERT mice were purchased from The Jackson Laboratory (USA). FTO^*fl/fl*^ mice were provided by Dr. Jun Gong’s lab (Tongji Hospital, Tongji Medical College, Huazhong University of Science and Technology, China). FTO^*fl/fl*^Col2a1-CreERT (FTO cKO) mice were generated by crossing FTO^*fl/fl*^ mice with Col2a1-CreERT mice; littermate FTO^*fl/fl*^ mice were used as control mice (FTO Ctrl). FTO cKO and FTO Ctrl mice were intraperitoneally injected with 30 mg/kg tamoxifen (T137974, Aladdin, Shanghai, China) dissolved in corn oil for 5 consecutive days at 4 weeks of age. Both FTO Ctrl and FTO cKO mice were fed with NCD or HFD for 4 weeks from 8 weeks of age and were subjected to DMM or sham surgery at 12 weeks of age (*n*=7−9 each group); then the mice were continued to be fed with NCD or HFD for another 8 weeks and were sacrificed at 20 weeks of age. For Ad-PDP2 articular injection, FTO cKO mice were intraperitoneally injected with 30 mg/kg tamoxifen for 5 consecutive days at 4 weeks of age; 8-week-old FTO cKO male mice were fed with HFD for 4 consecutive weeks, then were subjected to sham or DMM surgery at 12 weeks of age. Mice were continued on HFD for another 8 weeks after surgery; Ad-Ctrl and Ad-PDP2 viruses [10 µl of Ad-Ctrl and Ad-PDP2 adenoviruses (1×10^9^ PFUs), Genechem] were particularly injected once a week following DMM or sham operation for 7 weeks (*n*=5 each group), and were sacrificed at 20 weeks old.

### 2.3 Primary chondrocytes culture

Knee joint cartilages from newborn mouse pups (5-day-old) were dissected to harvest the primary chondrocytes. After being digested with 0.25% trypsin for 30 min, cartilage was cut into small pieces, puriﬁed and digested with 0.25% collagenase II dissolved in Dulbecco’s Modiﬁed Eagle Medium (DMEM)/F12 (C11330500BT, Gibco, Waltham, USA) culture medium at 37 °C for 6−8 h. The released chondrocytes were resuspended and cultured in DMEM/F12 supplemented with 10% fetal bovine serum (FBS) (FBS-S500, NEWZERUM, Christchurch, New Zealand), 100 U/ml penicillin, and 100 µg/ml streptomycin at 37 °C in an incubator. Cells were plated onto 10 cm plates and grown to approximately 80%−90% conﬂuence and passaged to 6-well plates before each experiment.

### 2.4 Small interfering RNA and plasmid transfections

Transfections were performed using Lipofectamine 3000 reagent. Chondrocytes were plated onto 6-well plates. For siRNA transfection, 50 pmol/L siRNA (RiboBio, Guangzhou, Guangdong, China) or scrambled siRNAs, 5 µl of Lipofectamine 3000, 250 µl of Opti-MEM, and 2 ml of complete medium were mixed and added to each well. The siRNA (sequence: FTO, 5’-CAACAGGCACCTTGGATTA-3’; PDP2, 5’-CCCTCAACATCTACCAGTT-3’; YTHDF2, 5’-GCATCAGTAGGGCAACAGA-3’) targeting mouse FTO, PDP2, and YTHDF2, and the relative scrambled siRNA negative control (si-NC) were purchased from RiboBio (Guangzhou, Guangdong, China). For FTO, PDP2, and YTHDF2 overexpression, chondrocytes were transfected with 1 µg overexpression plasmids in 2 ml complete medium, added with 5 µl Lipofectamine 3000 and 250 µl Opti-MEM. After 48 h of transfection, chondrocytes were harvested and subjected to diﬀerent treatments. The eﬃciency of knockdown and overexpression was conﬁrmed by quantitative reverse transcription polymerase chain reaction (qPCR) and Western blotting. FTO and PDP2 plasmids (p-FTO, p-PDP2) were cloned by Zhuandao Bio (Wuhan, Hubei, China). Based on methylated RNA immunoprecipitation (MeRIP) data, there are 5 predicted sites (m^6^A peaks) located at *Pdp2* mRNA exon 2. We mutated the first m^6^A site (base “A” to “T”, A1256T) to disrupt the effective m^6^A site. YTHDF2 plasmids (p-YTHDF2) were from Dr. Ke Chen’s lab (Tongji Hospital, Tongji Medical College, Huazhong University of Science and Technology, China).

### 2.5 Enzyme-linked immunosorbent assay, histopathological, and immunohistochemistry assay

After sacrificing the mice, the blood samples were collected, and serum levels of triglyceride [enzyme-linked immunosorbent assay (ELISA) kit, S0219S, Beyotime, Shanghai, China] and IL-1β (ELISA kit, EK0411, Boster, Wuhan, China) were measured in NCD, HFD, and HFD+DMM FTO cKO or FTO Ctrl mice. The knee tissues were decalciﬁed in 10% EDTA (pH 7.4) for 4 weeks. Subsequently, the tissues were embedded in paraﬃn and sectioned continuously at 5 µm thickness. For Safranin O/Fast Green (G1053, ServiceBio, Wuhan, Hubei, China), Boron-dipyrromethene (BODIPY), and Terminal deoxynucleotidyl transferase dUTP nick end labeling ‌(TUNEL, HY-K1079, MedChemExpress, Shanghai, China) staining, slices were deparaﬃnized and hydrated, incubated with Safranin O and Fast Green solution, or BODIPY and TUNEL probes following manufacturer’s instructions. For Safranin O staining, sections were incubated with Safranin O solution for 5 min. For BODIPY and TUNEL staining, sections or chondrocytes were fixed and incubated with BODIPY probes for 2−4 h, then counterstained with DAPI. Finally, all images were captured using a ﬂuorescence microscope.

The images were taken and scored for degree of articular cartilage damage according to the Osteoarthritis Research Association (OARSI) histopathology scoring system in a blind manner [28]. Synovitis was scored around the synovium tissue via previously reported methods [29]. For immunohistochemistry (IHC) staining, sections were deparaﬃnized, hydrated, and blocked with 5% bovine serum albumin (BSA) for 1 h at room temperature. After incubation with primary antibodies against matrix metalloproteinase (MMP) 13 (1:100, 18165-1-AP, Protentech, Wuhan, China), FTO (1:100, 27226-1-AP, Proteintech, Wuhan, China), PDP2 (1:200, A17190, Abclonal, Wuhan, China), Collagen II (1:200, sc-52658, Santa Cruz, Texas, USA), Aggrecan (1:100, 13880-1-AP, Proteintech, Wuhan China) overnight at 4 °C, sections were incubated with HRP-conjugated secondary antibodies (Jackson Lab, New York, USA) and counterstained with hematoxylin. Finally, all the images were taken under a microscope (EVOS AUTO, Invitrogen, Waltham, MA, USA).

### 2.6 Immunoﬂuorescence

The expression of collagen II or MMP13 in chondrocytes was performed by immunoﬂuorescence staining. After being ﬁxed in 4% paraformaldehyde and permeabilized in 0.1% Triton X-100, chondrocytes were incubated with primary antibodies against Collagen II (1:100, sc-52658, Santa Cruz, Texas, USA) and MMP13 (1:100, 18165-1-AP, Protentech, Wuhan, China) overnight at 4 °C, subsequently incubated with anti-mouse or anti-rabbit secondary antibodies for 1 h and counterstained with 4',6-diamidino-2-phenylindole (DAPI, AR1177, Boster, Wuhan, China). More details are listed in **Additional file 1: Methods**.

### 2.7 MeRIP sequencing (MeRIP-Seq) and data analysis

Total RNA from primary mouse chondrocytes transfected with si-NC and si-FTO was extracted using TRIzol reagent (9108, TaKaRa, Japan) in three independent replicates per group. Then, m^6^A sequencing was performed (Shanghai Jiayin Biotechnology Ltd., Shanghai, China). mRNA was fragmented and incubated with m^6^A antibody for immunoprecipitation. Immunoprecipitated RNA was analyzed through high-throughput sequencing. MeRIP-Seq was performed as described previously [30], [31]. In brief, total RNA was isolated and fragmented into approximately 100-nucleotide-long fragments. Approximately 5% of fragmented RNA was used as input RNA; the remaining RNA was analyzed by immunoprecipitation using affinity-purified anti-m^6^A polyclonal antibodies (ABE572, Millipore, Germany). Sequencing was carried out using an Illumina NovaSeq 6000 platform.

### 2.8 Statistical analysis

Statistical analyses were conducted using GraphPad Prism v. 5.0 (Graphpad Software Inc., San Diego, CA, USA). All experiments were independently repeated at least three times. One-way ANOVA followed by LSD’s post hoc tests were used to test the differences among more than two groups. The Student’s *t*-test was used to assess statistical differences in the data between two groups. Quantitative data were presented as mean±standard error of the mean (SEM). Values of *P*<0.05 were considered statistically signiﬁcant.

## 3 Results

### 3.1 FTO expression was downregulated in OA articular chondrocytes

**Fig. 1:**
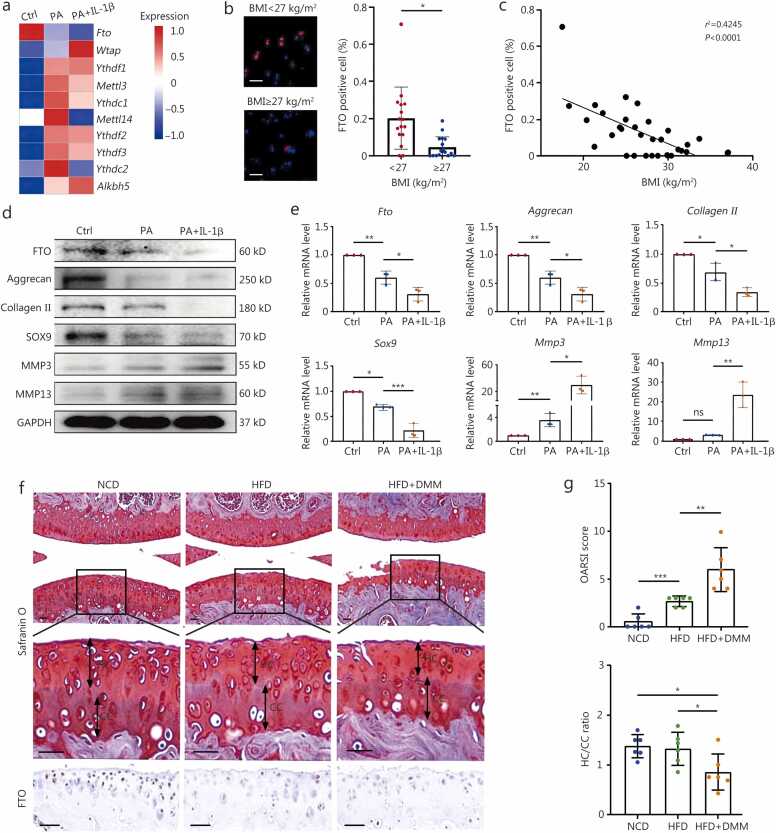
Reduced FTO expression in human OA samples, HFD and HFD+DMM animal models of OA, and *in vitro* mouse chondrocytes. **a** Heatmap showing changes in the mRNA abundance of m^6^A-related genes in chondrocytes stimulated with Ctrl, 200 μmol/L PA, or PA+5 ng/ml IL-1β for 24 h. **b** FTO staining and quantitative analysis of OA patients with BMI <27 kg/m^2^ and BMI ≥^2^7 kg/m^2^. **c** Correlation between FTO level in chondrocytes and BMI. Correlation equation: y=–0.01986x+0.6628. **d** Western blotting showing FTO, Aggrecan, Collagen Ⅱ, SOX9, MMP3, and MMP13 levels after treatment with PA and PA+IL-1β for 24 h in primary mouse chondrocytes. **e** Relative mRNA levels of *Fto*, *Aggrecan*, *Collagen Ⅱ*, *Sox9*, *Mmp3*, and *Mmp13* treated with 200 μmol/L PA or PA+5 ng/ml IL-1β for 24 h (*n*=3). **f** Safranin O staining and FTO IHC staining of the knee joint of NCD, HFD, and HFD+DMM mice (scale bar=200 μm). **g** OARSI score and HC/CC ratio. The data are presented as mean±SEM. ^⁎^*P*<0.05, ^⁎⁎^*P*<0.01, ^⁎⁎⁎^*P*<0.001, ns non-significant. FTO. Fat mass and obesity associated gene; Wtap. Wilms tumor 1-associated protein; Ythdf1. YTH N^6^-methyladenosine RNA binding protein 1; Mettl3. Methyltransferase-like 3; Ythdc1. YTH domain-containing protein 1; Mettl14. Methyltransferase-like 14; Ythdf2. YTH N^6^-methyladenosine RNA binding protein 2; Ythdf3. YTH N^6^-methyladenosine RNA binding protein 3; Ythdc2. YTH domain-containing protein 2; Alkbh5. AlkB homologue 5; NCD. Normal chow diet; HFD. High-fat diet; DMM. Destabilized medial meniscus; Ctrl. Control; PA. Palmitic acid; IL-1β. Interleukin-1β; BMI. Body mass index; Aggrecan. Aggregating proteoglycan; Collagen Ⅱ. Type II collagen; SOX9. Sex determining region Y-box 9; MMP3. Matrix metalloproteinase 3; MMP13. Matrix metalloproteinase 13; GAPDH. *Glyceraldehyde-3-phosphate dehydrogenase;* OARSI. Osteoarthritis Research Association; HC/CC ratio. Hyaline cartilage-to-calcified cartilage ratio; IHC. Immunohistochemistry; SEM. Standard error of the mean.

CCK-8 assay showed that chondrocytes with PA treatment resulted in comparable cell viability vs. control (Ctrl) cells; PA+IL-1β exposure exhibited a decline in cell viability but did not reach a significant difference (**Additional file 1:** Fig. S1a). RNA-Seq analysis confirmed that among all components of the m^6^A writer complex, only the *FTO* mRNA was downregulated in chondrocytes treated both with PA alone and with PA+IL-1β (Fig. 1**a**). *De novo* lipogenesis (DNL) and FA oxidation, which are known to regulate the breakdown and synthesis of FAs, are important determinants of lipid accumulation [32], [33]. Following PA treatment, the expression of key DNL genes, including acetyl-coenzyme A carboxylase alpha (*Acaca*), fatty acid synthase (*Fasn*), stearoyl-CoA desaturase (*Scd*)1, acetyl-CoA synthetase (*Acss*)1, and *Acss2*, was downregulated (**Additional file 1:** Fig. S1b). Pyruvate metabolism is a critical process in the generation of acetyl-CoA. PA treatment also reduced the expression of *Pdp1*, *Pdp2*, pyruvate kinase (*Pdk*)*1*, *Pdk2*, and *Pdk3*, but not *Pdk4*, as well as the expression of lactate dehydrogenase (*Ldhb*)*.* When IL-1β was co-stimulated with PA to exacerbate inflammation, the decreases in *Accs2*, *Scd1*, *Ldhb*, and *Pdk3* became more pronounced. In contrast, the expression of acyl-CoA thioesterases (*Acot1*, *Acot2*, *Acot4*, *Acot6*, *Acot9*) was elevated significantly after PA exposure, suggesting that PA disrupts multiple aspects of lipid metabolism and hydrolysis. Moreover, PA and IL-1β co-stimulation remarkably exacerbated inflammation and further increased *Acot2*, *Acot4*, *Acot6*, and *Acot9* levels (**Additional file 1:** Fig. S1b). Using clinical cartilage samples, FTO staining demonstrated a significantly lower FTO expression in obese patients with BMI ≥27 kg/m^2^, and revealed a significant negative correlation between FTO expression and BMI (Fig. 1**b, c**). Subsequently, we investigated the roles of PA alone and PA+IL-1β, respectively, in OA pathogenesis using mouse primary chondrocytes. FTO, as a fat mass and obesity-associated protein, exhibited a gradually declining trend after treatment with both PA alone and PA+IL-1β (Fig. 1**d, e; Additional file 1:** Fig. S1c**)**. PA treatment significantly reduced the expression of Aggrecan, Collagen Ⅱ, and SOX9, three collagen-related molecules, while notably increasing the expression of MMP3 and MMP13. IL-1β was shown to further exacerbate the effects of PA, as evidenced by even lower levels of Aggrecan and Collagen Ⅱ, and higher levels of MMP3 and MMP13 after PA+IL-1β co-stimulation vs. PA treatment alone (Fig. 1**d, e; Additional file 1:** Fig. S1c). Consistently, in the HFD-fed mouse model (12 weeks of HFD feeding, **Additional file 1:** Fig. S1d), we observed a robust increase in body weight and serum glucose levels (**Additional file 1:** Fig. S1e). The HFD-fed mice exhibited mild-to-moderate destruction and erosion of superficial cartilage, whereas HFD feeding combined with DMM surgery resulted in far more severe cartilage destruction and higher OARSI scores (Fig. 1**f, g**). The ratio of hyaline cartilage to calcified cartilage (HC/CC) reflects a superficial cartilage proportion, which negatively correlates with the severity of cartilage destruction [34]. In our experiment, the HC/CC ratio showed no obvious changes in HFD-fed mice but was downregulated in HFD+DMM mice (Fig. 1**g**). Moreover, the number of FTO positive chondrocytes in the cartilage was remarkably reduced in HFD-fed mice, and this reduction became more pronounced in HFD+DMM mice (Fig. 1**f; Additional file 1:** Fig. S1f).

### 3.2 Genetically, adenoviruses, or pharmacologically induced FTO inhibition aggravated HFD- and HFD+DMM-induced cartilage degeneration

**Fig. 2:**
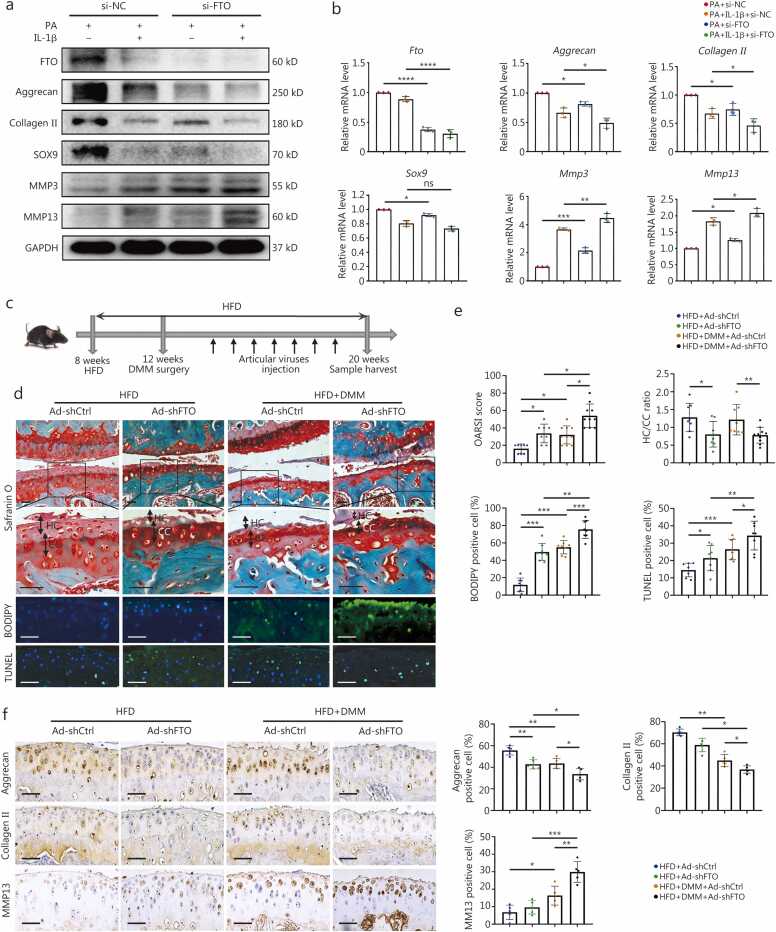
*Fto* knockdown promotes cartilage degeneration in HFD and HFD+DMM models. **a** Representative Western blotting showing FTO, Aggrecan, Collagen Ⅱ, SOX9, MMP3, and MMP13 expression after treatment with PA and PA+IL-1β in primary mouse chondrocytes with or without *Fto* knockdown. **b** Relative mRNA levels of *Fto*, *Aggrecan*, *Collagen Ⅱ*, *Sox9*, *Mmp3*, and *Mmp13* after treatment with PA and PA+IL-1β in primary mouse chondrocytes with or without *Fto* knockdown. **c** Schematic illustration of the experimental design of HFD and HFD+DMM mice with Ad-shCtrl or Ad-shFTO weekly injection. **d** Safranin O, BODIPY, and TUNEL staining of the knee joint of HFD and HFD+DMM mice injected with Ad-shCtrl or Ad-shFTO viruses (scale bar=200 μm). **e** OARSI score, HC/CC ratio, BODIPY positive chondrocytes, and TUNEL positive chondrocytes in HFD and HFD+DMM mice injected with Ad-shCtrl or Ad-shFTO viruses. **f** Aggrecan, Collagen Ⅱ, and MMP13 positive cells in HFD and HFD+DMM mice injected with Ad-shCtrl or Ad-shFTO viruses (scale bar=200 μm). The data are presented as mean±SEM. ^⁎^*P*<0.05, ^⁎⁎^*P*<0.01, ^⁎⁎⁎^*P*<0.001, ^⁎⁎⁎⁎^*P*<0.0001, ns non-significant. si-NC. Small interfering RNA-negative control; si-FTO. Small interfering RNA-FTO; FTO. Fat mass and obesity associated gene; HFD. High fat diet; DMM. Destabilized medial meniscus; PA. Palmitic acid; IL-1β. Interleukin-1β; Aggrecan. Aggregating proteoglycan; Collagen Ⅱ. Type II collagen; SOX9. Sex determining region Y-box 9; MMP3. Matrix metalloproteinase 3; MMP13. Matrix metalloproteinase 13; GAPDH. *Glyceraldehyde-3-phosphate dehydrogenase;* Ad-shCtrl. Adenovirus-short hairpin control; Ad-shFTO. Adenovirus-short hairpin FTO; BODIPY. Boron-dipyrromethene; TUNEL. Terminal deoxynucleotidyl transferase dUTP nick end labeling; OARSI. Osteoarthritis Research Association; HC/CC ratio. Hyaline cartilage-to-calcified cartilage ratio; SEM. Standard error of the mean.

To further confirm the involvement of FTO in OA pathogenesis, primary mouse chondrocytes were transfected with si-FTO; the knockout efficiency of si-FTO *in vitro* over time was tested in **Additional file 1:** Fig. S2a**, b**. Results showed that *Fto* knockdown significantly increased MMP3 and MMP13 levels and reduced Aggrecan and Collagen Ⅱ levels both in PA and PA+IL-1β-treated chondrocytes, while SOX9 level only decreased in PA-exposed chondrocytes (Fig. 2**a, b; Additional file 1:** Fig. S2c). Meanwhile, Collagen Ⅱ immunostaining demonstrated cartilage loss following *Fto* knockdown (**Additional file 1:** Fig. S2d). Lipid accumulation in chondrocytes was visualized using BODIPY, a fluorescent probe for neutral lipids; our findings indicated that PA treatment alone increased lipid deposition while PA+IL-1β co-stimulation further aggravated this effect. Moreover, *Fto* knockdown notably increased lipid deposition both in PA- and PA+IL-1β-treated chondrocytes (**Additional file 1:** Fig. S2e). Conversely, FTO overexpression (p-FTO) upregulated Aggrecan, Collagen Ⅱ, and SOX9 levels, and downregulated MMP3 and MMP13 levels in PA+IL-1β-treated chondrocytes (**Additional file 1:** Fig. S2f**, g**). Additionally, *Fto* knockdown activated inflammatory responses as evidenced by elevated p-P65 levels; in contrast, FTO overexpression reduced p-P65 levels (**Additional file 1:** Fig. S3a**, b**), emphasizing the involvement of FTO in inflammation regulation as a vital driver of OA. To further elucidate the role of FTO *in vivo*, we injected Ad-shFTO viruses into the knee joint cavity of HFD-fed wild-type mice (Fig. 2**c**); the knockout efficiency of Ad-shFTO *in vitro* over time was also tested in **Additional file 1:** Fig. S3c**, d**. IHC staining showed the knockout efficiency of FTO in the cartilage (**Additional file 1:** Fig. S3e). It was found that HFD induced mild cartilage damage in mice, and *Fto* knockdown markedly aggravated cartilage erosion as evidenced by higher OARSI scores and a lower HC/CC ratio (Fig. 2**d, e**). Increased BODIPY and TUNEL positive chondrocytes were also observed after *Fto* knockdown in HFD-fed mice (Fig. 2**d, e**). Meanwhile, the Aggrecan levels were downregulated, whereas Collagen Ⅱ and MMP13 expression remained unchanged in Ad-shFTO-intervened HFD mice (Fig. 2**f**). Since DMM surgery was shown to aggravate cartilage degeneration and lipid deposition, we further established DMM models using HFD-fed mice. It was found that, compared to Ad-shCtrl-intervened mice, Ad-shFTO infection significantly aggravated cartilage degeneration, evidenced by higher OARSI scores and more severe synovitis (Fig. 2**d; Additional file 1:** Fig. S3f). Decreased HC/CC ratio, increased OARSI score, BODIPY positive chondrocytes, and TUNEL positive chondrocytes, and less Aggrecan, Collagen Ⅱ, as well as more MMP13 positive chondrocytes, were found in Ad-shFTO-treated HFD+DMM mice compared with Ad-Ctrl-injected HFD+DMM mice (Fig. 2**d-f**).

FB23 is a selective FTO inhibitor [35]. The inhibitory effect of FB23 was further validated, with the results shown in **Additional file 1:** Fig. S4a**, b**. FB23 treatment also led to a decline in SOX9 levels in PA-treated but not in IL-1β+PA co-treated chondrocytes, but it only affected Aggrecan and Collagen Ⅱ in PA-treated chondrocytes, whereas the MMP3 and MMP13 levels remained unchanged (**Additional file 1:** Fig. S4c**, d**). These results suggest that FTO inhibition can aggravate the degeneration of chondrocytes in OA pathogenesis. However, a recent study reported that FB23-2, a FB23 analogue, exerts an off-target inhibitory effect on human dihydroorotate dehydrogenase (DHODH), independent of FTO [36]. Therefore, we investigated the effect of FB23 on mouse DHODH and found that FB23, similar to FB23-2, also interacts with DHODH. Since FB23 does not affect the protein expression of DHODH, we measured DHODH activity via ELISA and confirmed that the DHODH activity was notably inhibited by FB23 (**Additional file 1:** Fig. S4e**, f**), to rule out the role of DHODH in OA, we used specific DHODH inhibitor Brequinar (Breq) [37] to treat the chondrocytes and DHODH activity was significantly reduced but did not affect Aggrecan, Collagen Ⅱ, MMP3 or MMP13 expression after PA and PA+IL-1β exposure (**Additional file 1:** Fig. S4f**, g**), indicating FB23 exacerbates OA mainly through targeting FTO. These results suggest that FTO inhibition can aggravate the degeneration of chondrocytes in OA pathogenesis.

FB23 was administered to investigate the effects of pharmacological FTO inhibition on cartilage degeneration (**Additional file 1:** Fig. S4h). IHC staining demonstrated that intra-articular administration of FB23 resulted in a decreased number of FTO positive chondrocytes (**Additional file 1:** Fig. S4i). While FB23 injection did not affect cartilage degeneration in HFD-fed mice, it significantly exacerbated cartilage damage in HFD+DMM mice, evidenced by a higher OARSI score, HC/CC ratio, and larger numbers of BODIPY and TUNEL positive cells (**Additional file 1:** Fig. S4j**, k**). Additionally, synovitis was notably enhanced in FB23-treated HFD+DMM mice (**Additional file 1:** Fig. S4l). Furthermore, FB23 treatment reduced Aggrecan and Collagen Ⅱ levels in HFD+DMM mice but not in HFD-fed mice, while showing no effect on MMP13 expression (**Additional file 1:** Fig. S4m).

**Fig. 3:**
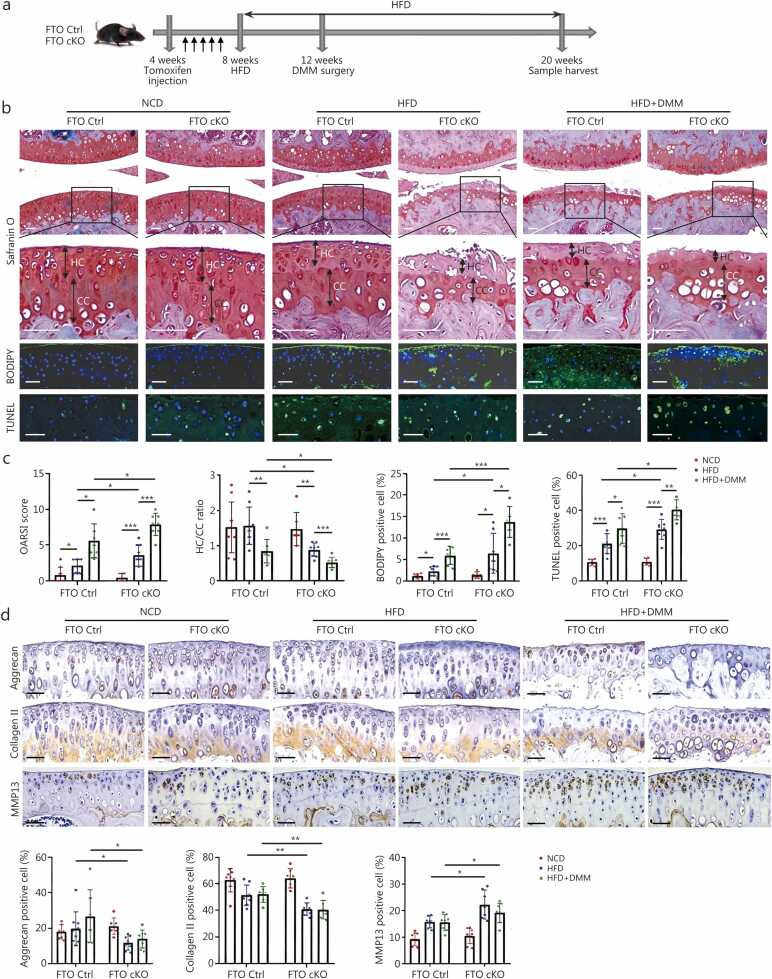
Deletion of *Fto* in mouse chondrocytes promoted cartilage destruction in HFD and HFD+DMM mice. **a** Schematic illustration of tamoxifen administration, HFD and HFD+DMM experimental design for FTO Ctrl and FTO cKO mice. **b** Safranin O, BODIPY, and TUNEL staining of the knee joint of NCD, HFD, and HFD+DMM mice in FTO Ctrl and FTO cKO mice (scale bar=200 µm). **c** OARSI score, HC/CC ratio, BODIPY positive cells, and TUNEL positive cells of NCD, HFD, and HFD+DMM mice in FTO Ctrl and FTO cKO mice. **d** IHC staining of Aggrecan, Collagen II, and MMP13 in FTO Ctrl and FTO cKO NCD, HFD, and HFD+DMM mice cartilage (scale bar=200 µm) (*n*=7−9). The data are presented as mean±SEM. ^⁎^*P*<0.05, ^⁎⁎^*P*<0.01, ^⁎⁎⁎^*P*<0.001. FTO. Fat mass and obesity associated gene; HFD. High fat diet; DMM. Destabilized medial meniscus; Ctrl. Control; cKO. conditional knockout; NCD. Normal chow diet; IHC. Immunohistochemistry; Collagen II. Type II collagen; Aggrecan. Aggregating proteoglycan; MMP13. Matrix metalloproteinase 13; BODIPY. Boron-dipyrromethene; TUNEL. Terminal deoxynucleotidyl transferase dUTP nick end labeling; OARSI. Osteoarthritis Research Association; HC/CC ratio. Hyaline cartilage-to-calcified cartilage ratio; SEM. Standard error of the mean.

To exclude the off-target effects of FTO inhibitors and adenoviruses, we established cartilage-specific FTO cKO mouse models to evaluate whether *FTO* depletion in cartilage recapitulates previous findings (see experimental design in Fig. 3**a**). FTO staining confirmed a reduced FTO level in the cartilage of FTO cKO mice (**Additional file 1:** Fig. S5a). The body weight and blood glucose levels were not significantly altered in both FTO cKO mice and FTO Ctrl NCD-fed mice, but were notably reduced in HFD-fed FTO cKO mice (**Additional file 1:** Fig. S5b**, c**), demonstrating that *Fto* knockout in cartilage has similar effects on weight control and circulating glucose in global and adipose FTO cKO mice [38], [39]. Surprisingly, DMM surgery abated the difference in body weight between groups, while FTO cKO mice still exhibited lower blood glucose levels (**Additional file 1:** Fig. S5d). *Fto* knockout led to higher OARSI scores in both HFD-fed and HFD+DMM mice but not in NCD-fed mice. Meanwhile, the HC/CC ratio was downregulated in both HFD-fed and HFD+DMM FTO cKO mice compared to FTO Ctrl mice. BODIPY staining revealed that both HFD-fed and HFD+DMM FTO cKO mice had increased lipid deposition and a larger number of TUNEL positive chondrocytes (Fig. 3**b, c**). We measured triglyceride and IL-1β levels after sacrificing the mice. Unlike FTO Ctrl mice, FTO cKO mice didn’t show increased triglyceride levels after HFD and HFD+DMM, and had decreased triglyceride levels compared with FTO Ctrl mice after HFD+DMM; these findings may be attributed to increased lipid deposition in organs. However, IL-1β levels were not changed between the two genotypes, suggesting local hyperinflammation in FTO cKO mice (**Additional file 1:** Fig. S5e). Additionally, FTO cKO mice had higher synovitis scores (**Additional file 1:** Fig. S5f), accompanied by reduced Aggrecan and Collagen Ⅱ levels and elevated MMP13 expression (Fig. 3**d**).

### 3.3 Identification of FTO-mediated targets by MeRIP-Seq

**Fig. 4:**
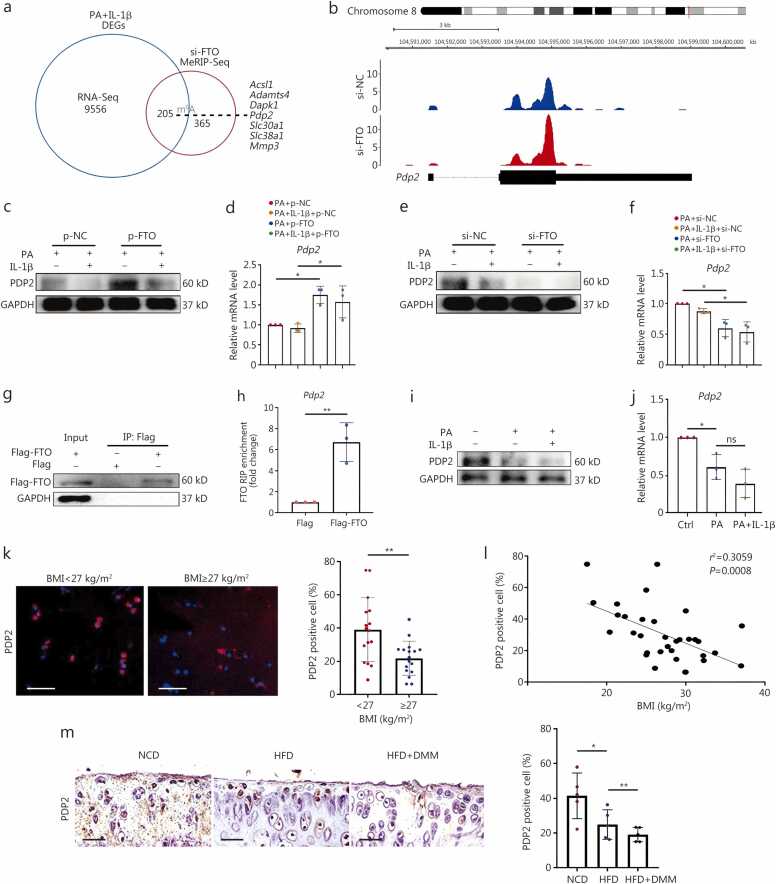
MeRIP-Seq and RNA-Seq identified *Pdp2* as a potential target of FTO-mediated m^6^A modification. **a** The intersection of DEGs identified by PA+IL-1β (200 μmol/L PA+5 ng/ml IL-1β treatment for 24 h) treated chondrocytes and m^6^A methylation genes in *Fto* knockdown chondrocytes. **b** The m^6^A peaks on *Pdp2* mRNA transcripts in si-NC and si-FTO-transfected chondrocytes showing *Pdp2* m^6^A peaks significantly increased. **c** Representative Western blotting showing PDP2 expression after treatment with PA and PA+IL-1β in primary mouse chondrocytes with or without FTO overexpression. **d***Pdp2* mRNA level after treatment with PA and PA+IL-1β in primary mouse chondrocytes with or without FTO overexpression. **e** Representative Western blotting showing PDP2 expression after treatment with PA and PA+IL-1β in primary mouse chondrocytes with or without *Fto* knockdown. **f***Pdp2* mRNA level after treatment with PA and PA+IL-1β in primary mouse chondrocytes with or without *Fto* knockdown. **g** Co-immunoprecipitation showing FTO was successfully immunoprecipitated after Flag-FTO overexpression in the presence of 200 μmol/L PA and 5 ng/ml IL-1β. **h** RIP assays showing FTO interacted with *Pdp2* mRNA. **i** Representative Western blotting showing PDP2 expression after treatment with PA and PA+IL-1β in primary mouse chondrocytes. **j***Pdp2* mRNA level after treatment with PA and PA+IL-1β in primary mouse chondrocytes. **k** PDP2 staining in OA patients with BMI <27 kg/m^2^ and BMI ≥^2^7 kg/m^2^ and quantitative analysis of PDP2 positive cells (scale bar=200 µm). **l** Correlation between PDP2 level in chondrocytes and BMI. Correlation equation: y=–0.02003x+0.8473. **m** PDP2 positive cells in the knee joint of NCD, HFD, and HFD+DMM mice (scale bar=200 µm) (*n*=5). The data are presented as mean±SEM. ^⁎^*P*<0.05, ^⁎⁎^*P*<0.01, ns non-significant. Ctrl. Control; MeRIP. Methylated RNA immune-precipitation; DEGs. Differentially expressed genes; m^6^A. N^6^-methyladenine; Acsl1. Acyl-CoA synthetase 1; Adamts4. A disintegrin and metalloproteinase with thrombospondin motifs-4; Dapk1. Death-associated protein kinase 1; Slc30a1. Solute carrier family 30 member 1; Slc38a1. Solute carrier family 38 member 1; Mmp3. Matrix metalloproteinase 3; PDP2. Pyruvate dehydrogenase phosphatases2; FTO. Fat mass and obesity associated gene; PA. Palmitic acid; IL-1β. Interleukin-1β; si-NC. Small interfering RNA-negative control; si-FTO. Small interfering RNA-FTO; p-NC. Plasmid negative control; p-FTO. Plasmid-FTO overexpression; GAPDH. Glyceraldehyde-3-phosphate dehydrogenase; RIP. RNA immunoprecipitation; BMI. Body mass index; NCD. Normal chow diet; HFD. High fat diet; DMM. Destabilized medial meniscus; SEM. Standard error of the mean.

To further identify FTO target genes, MeRIP-Seq was performed in *Fto* knockdown chondrocytes using si-FTO under PA+IL-1β co-stimulation, with three independent batches of replicates. Totally 365 screened altered m^6^A peaks (*P*<0.05) were found in MeRIP-Seq of si-FTO-transfected chondrocytes. There were 9556 regulated genes in RNA-Seq (log_2_ fold change>0, *P*<0.05) data of PA+IL-1β-treated chondrocytes after reanalyzing the data; 205 overlapped genes were identified (Fig. 4**a**). Among them, *Pdp2*, a gene involved in metabolic processes, was selected. Compared with si-NC chondrocytes, the chondrocytes transfected with si-FTO exhibited higher m^6^A peaks in the coding sequence (CDS) of *Pdp2* mRNA (Fig. 4**b**). We therefore speculated that FTO may contribute to cartilage loss by modulating the m^6^A level of *Pdp2*.

To confirm the sequencing findings, we further verified that FTO overexpression increased the protein and mRNA levels of *PDP2* in both PA and PA+IL-1β-treated chondrocytes (Fig. 4**c, d; Additional file 1:** Fig. S6a), whereas *Fto* knockdown yielded exactly opposite outcomes (Fig. 4**e, f; Additional file 1:** Fig. S6b). Similar results were obtained in FB23-treated chondrocytes (**Additional file 1:** Fig. S6c**, d**). Additionally, PDP2 expression was also reduced in NCD-fed and HFD-fed FTO cKO mice compared to FTO Ctrl mice but not in HFD+DMM mice (**Additional file 1:** Fig. S6e). Consistently, the RIP assay results revealed that FTO interacted with *Pdp2* mRNA (Fig. 4**g, h**), and FB23 treatment weakened the binding between FTO and *Pdp2* mRNA (**Additional file 1:** Fig. S6f**, g**), indicating that *Pdp2* is a direct target of FTO.

### 3.4 PDP2 was involved in cartilage degeneration

**Fig. 5:**
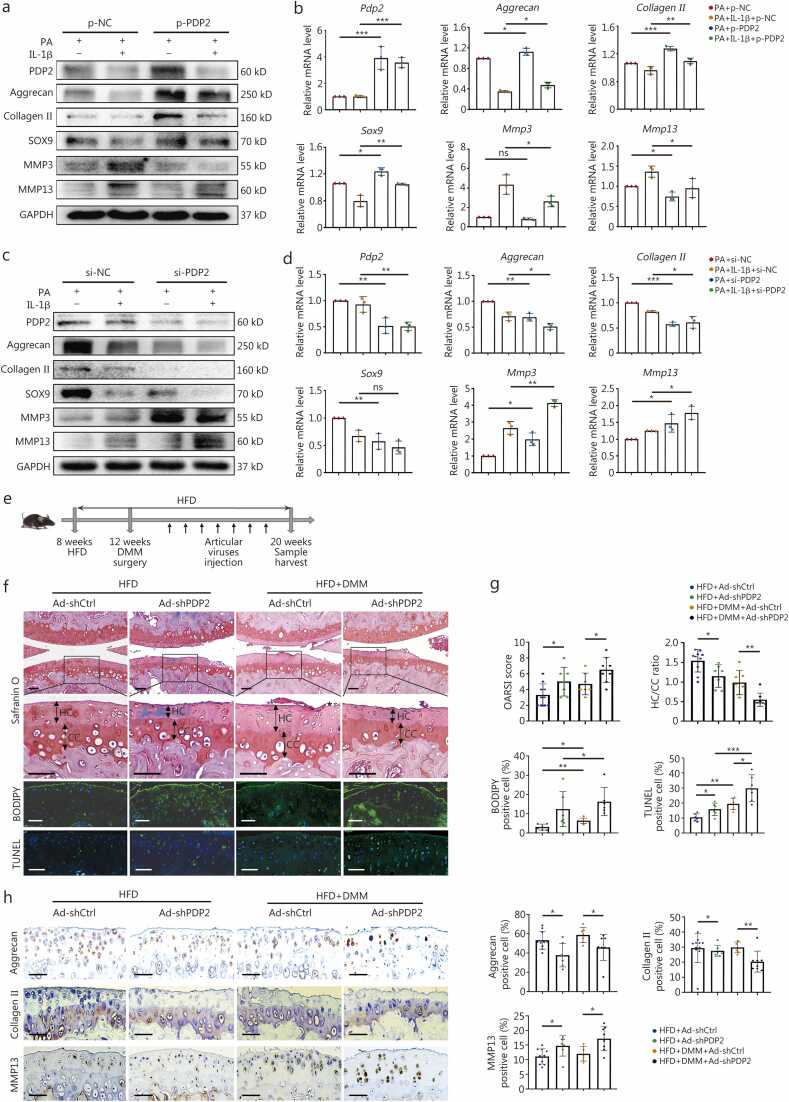
PDP2 modulates cartilage destruction in HFD and HFD+DMM mice. **a** Representative Western blotting showing PDP2, Aggrecan, Collagen Ⅱ, SOX9, MMP3, and MMP13 expression after treatment with PA and PA+IL-1β in primary mouse chondrocytes with or without PDP2 overexpression. **b***Pdp2*, *Aggrecan*, *Collagen Ⅱ*, *Sox9*, *MMP3*, and *MMP13* mRNA levels after treatment with PA and PA+IL-1β in primary mouse chondrocytes with or without PDP2 overexpression. **c** Representative Western blotting showing PDP2, Aggrecan, Collagen Ⅱ, SOX9, MMP3, and MMP13 expression after treatment with PA and PA+IL-1β in primary mouse chondrocytes with or without *Pdp2* knockdown. **d***Pdp2*, *Aggrecan*, *Collagen Ⅱ*, *Sox9*, *Mmp3*, and *Mmp13* mRNA levels after treatment with PA and PA+IL-1β in primary mouse chondrocytes with or without *Pdp2* knockdown. **e** Schematic illustration of the experimental design of HFD and HFD+DMM mice with Ad-shCtrl or Ad-shPDP2 weekly injection. **f** Safranin O, BODIPY, and TUNEL staining of the knee joint of HFD and HFD+DMM mice in Ad-shCtrl or Ad-shPDP2-injected mice. **g** OARSI score, HC/CC ratio, BODIPY positive cells, and TUNEL positive cells in HFD and HFD+DMM mice injected with Ad-shCtrl or Ad-shPDP2 viruses (*n*=6-10). **h** IHC staining of Aggrecan, Collagen II, and MMP13 in HFD and HFD+DMM mice injected with Ad-shCtrl or Ad-shPDP2 viruses (scale bar=200 µm) (*n*=6−10 each group, *n*=6 for BODIPY and TUNEL staining). The data are presented as mean±SEM. ^⁎^*P*<0.05, ^⁎⁎^*P*<0.01, ^⁎⁎⁎^*P*<0.001, ns non-significant. PDP2. Pyruvate dehydrogenase phosphatases2; p-NC. Plasmid negative control; p-PDP2. Plasmid-PDP2 overexpression; si-NC. Small interfering RNA-negative control; si-PDP2. Small interfering RNA-PDP2; HFD. High fat diet; DMM. Destabilized medial meniscus; PA. Palmitic acid; IL-1β. Interleukin-1β; Aggrecan. Aggregating proteoglycan; Collagen Ⅱ. Type II collagen; SOX9. Sex determining region Y-box 9; MMP3. Matrix metalloproteinase 3; MMP13. Matrix metalloproteinase 13; GAPDH. *Glyceraldehyde-3-phosphate dehydrogenase;* Ad-shCtrl. Adenovirus-short hairpin control; Ad-shPDP2. Adenovirus-short hairpin PDP2; BODIPY. Boron-dipyrromethene; TUNEL. Terminal deoxynucleotidyl transferase dUTP nick end labeling; OARSI. Osteoarthritis Research Association; HC/CC ratio. Hyaline cartilage-to-calcified cartilage ratio; IHC. Immunohistochemistry; SEM. Standard error of the mean.

The results above prompted us to further explore the role of PDP2 in OA degeneration. We treated chondrocytes with PA and found that PA significantly reduced PDP2 expression, while co-stimulation with IL-1β further intensified this effect, although not reaching a significant difference (Fig. 4**i, j; Additional file 1:** Fig. S6h). PDP2 staining showed that PDP2 expression was significantly lower in obese patients (BMI ≥27 kg/m^2^), indicating a negative correlation with BMI (Fig. 4**k, l**). Additionally, the findings above revealed that PDP2 expression was reduced in HFD-fed mice (**Additional file 1:** Fig. S6e). Notably, it also showed a decline in HFD+DMM mice but did not reach a significant difference, which is consistent with *in vitro* results (Fig. 4**m**). Moreover, PDP2 overexpression increased Aggrecan, Collagen Ⅱ, and SOX9 levels in both PA and PA+IL-1β-treated chondrocytes, whereas the MMP3 and MMP13 levels were downregulated (Fig. 5**a, b; Additional file 1:** Fig. S6i). Consistently, *Pdp2* knockdown with si-PDP2 notably reduced Aggrecan, Collagen Ⅱ, and SOX9 levels but increased MMP3 and MMP13 levels (Fig. 5**c, d; Additional file 1:** Fig. S6j).

To investigate the *in vivo* role of PDP2, we injected Ad-shPDP2 viruses into the knee joint of HFD-fed and HFD+DMM wild-type mice, and then verified the knockout efficiency by IHC staining (Fig. 5**e; Additional file 1:** Fig. S6k). Safranin O staining demonstrated that Ad-shPDP2 treatment markedly aggravated cartilage erosion, evidenced by higher OARSI scores and reduced HC/CC ratios. Meanwhile, Ad-shPDP2 treatment also increased the numbers of BODIPY and TUNEL positive chondrocytes (Fig. 5**f, g**). IHC staining showed that the Aggrecan and Collagen Ⅱ levels were decreased, whereas the MMP13 level was increased (Fig. 5**h**). Furthermore, Ad-shPDP2 injection induced more severe synovitis both in HFD-fed and HFD+DMM mice (**Additional file 1:** Fig. S6l).

### 3.5 PDP2 reversed cartilage degradation induced by *Fto* knockdown

**Fig. 6:**
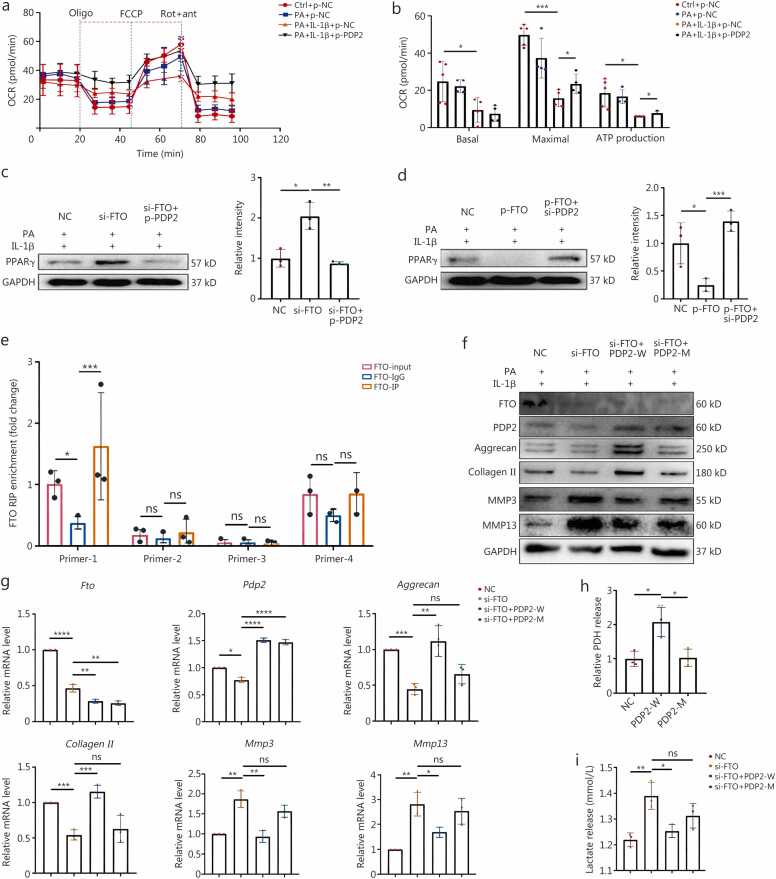
Overexpression of PDP2-W but not PDP2-M rescued *Fto* knockdown-induced cartilage degeneration. **a** OCR of chondrocytes following 24 h of PA and PA+IL-1β treatment with or without PDP2 overexpression was assessed before and after sequential treatment with oligomycin, FCCP, rotenone, and antimycin. **b** Basal and maximal OCR, and ATP production in cells shown in **a**. **c** PPARγ expression in chondrocytes transfected with NC, si-FTO, and si-FTO+PDP2. **d** PPARγ expression in chondrocytes transfected with NC, p-FTO, and p-FTO+si-PDP2. **e** FTO RIP enrichment in different m^6^A sites. **f** Representative Western blotting of FTO, PDP2, Aggrecan, Collagen Ⅱ, MMP3, and MMP13 after *Fto* knockdown in chondrocytes treated with PA+IL-1β with PDP2-W or PDP2-M overexpression. **g***Fto*, *Pdp2*, *Aggrecan*, *Collagen Ⅱ*, *Mmp3*, and *Mmp13* mRNA levels after *Fto* knockdown in chondrocytes treated with PA+IL-1β in chondrocytes with PDP2-W or PDP2-M overexpression. **h** PDH activity in chondrocytes transfected with PDP2-W and PDP2-M plasmids. **I** Lactate levels in the medium of si-FTO, PDP2-W, and PDP2-M transfected chondrocytes treated with PA+IL-1β. The data are presented as mean±SEM. ^⁎^*P*<0.05, ^⁎⁎^*P*<0.01, ^⁎⁎⁎^*P*<0.001, ^⁎⁎⁎⁎^*P*<0.0001, ns non-significant. Ctrl. Control; PDP2-W. Wild-type pyruvate dehydrogenase phosphatases 2; PDP2-M. Mutant pyruvate dehydrogenase phosphatases 2; FTO. Fat mass and obesity associated gene; OCR. Oxygen consumption rate; PA. Palmitic acid; IL-1β. Interleukin-1β; FCCP. Carbonyl cyanide-p-trifluoromethoxyphenylhydrazone; Rot. Rotenone; ant. Antimycin A; PPARγ. Peroxisome proliferator-activated receptor γ; si-NC. Small interfering RNA-negative control; si-FTO. Small interfering RNA-FTO; p-NC. Plasmid negative control; p-FTO. Plasmid-FTO overexpression; p-PDP2. Plasmid-PDP2 overexpression; si-NC. Small interfering RNA-negative control; si-PDP2. Small interfering RNA-PDP2; RIP. RNA immunoprecipitation; Aggrecan. Aggregating proteoglycan; Collagen Ⅱ. Type II collagen; MMP3. Matrix metalloproteinase 3; MMP13. Matrix metalloproteinase 13; PDH. Pyruvate dehydrogenase; SEM. Standard error of the mean.

We monitored oxygen consumption using Seahorse assays; PA-treated chondrocytes exhibited a decreased trend in basal and maximal OCR along with declined ATP production. PA+IL-1β significantly enhanced the difference; PDP2 overexpression, however, remarkably rescued the maximal OCR and ATP production decline induced by PA+IL-1β (Fig. 6**a, b**). Since FTO has been reported to downregulate the PPARγ pathway [40], we further investigated the role of PDP2 in regulating PPARγ. The results showed that si-FTO induced an increase in PPARγ levels, which was reversed by PDP2 overexpression (Fig. 6**c**), and vice versa (Fig. 6**d**). According to MeRIP-Seq results, the m^6^A peaks were located at the CDS region of *Pdp2* mRNA. Five sites (m^6^A peaks) located in exon 2 of *Pdp2* mRNA were predicted. We then designed 4 primers targeting these sites, with site 3 and site 4 merged into primer 3 due to their proximity. The m^6^A RIP analysis demonstrated that m^6^A was significantly enriched at site 1, while site 4 was slightly affected but did not reach a significant difference (Fig. 6**e**). To further clarify whether the m^6^A methylation at the CDS region is associated with alterations in PDP2, we introduced synonymous mutations at site 1 m^6^A site on the PDP2 CDS (A1256T). We then investigated whether FTO exerts its role through PDP2. Chondrocytes transfected with si-FTO showed decreased Aggrecan and Collagen Ⅱ, elevated MMP3 and MMP13 levels; these changes were significantly restored by PDP2 overexpression in PA+IL-1β-treated chondrocytes. Western blotting and qPCR results revealed that PDP2-W, but not PDP2-M (A1256T), could reverse the decrease in Aggrecan and Collagen Ⅱ levels, and the increase in MMP3 and MMP13 expression (Fig. 6**f, g; Additional file 1:** Fig. S6m). PDP2 is known to be positively correlated with PDH activity. We found that PDP2-W drastically upregulated PDH activity, whereas PDP2-M exhibited an opposite effect, indicating a loss of function in PDP2-M (Fig. 6**h**). Moreover, as an important product of lipid metabolism, lactate expression was increased in si-FTO-transfected chondrocytes and was significantly reversed by PDP2-W but not PDP2-M transfection (Fig. 6**i**).

**Fig. 7:**
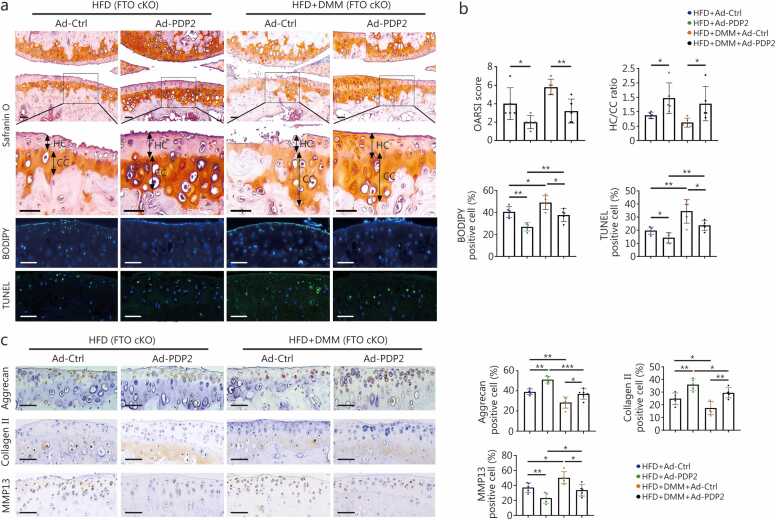
Overexpression of PDP2 rescued cartilage degradation in HFD-fed and HFD+DMM FTO cKO mice injected with Ad-Ctrl or Ad-PDP2 viruses. **a** Safranin O, BODIPY, and TUNEL staining of the knee joint of HFD and HFD+DMM mice in FTO cKO mice (scale bar=200 µm). **b** OARSI score, HC/CC ratio, BODIPY positive cells, and TUNEL positive cells in HFD and HFD+DMM FTO cKO mice injected with Ad-Ctrl or Ad-PDP2 viruses. **c** IHC staining of Aggrecan, Collagen II, and MMP13 in HFD and HFD+DMM FTO cKO mice injected with Ad-Ctrl or Ad-PDP2 viruses (scale bar=200 µm) (*n*=5). The data are presented as mean±SEM. ^⁎^*P*<0.05, ^⁎⁎^*P*<0.01, ^⁎⁎⁎^*P*<0.001. PDP2. Pyruvate dehydrogenase phosphatases 2; HFD. High fat diet; DMM. Destabilized medial meniscus; FTO. Fat mass and obesity associated gene; cKO. Conditional knockout; Ad-Ctrl. Adenovirus-control; Ad-PDP2. Adenovirus-PDP2 overexpression; BODIPY. Boron-dipyrromethene; TUNEL. Terminal deoxynucleotidyl transferase dUTP nick end labeling; OARSI. Osteoarthritis Research Association; HC/CC ratio. Hyaline cartilage-to-calcified cartilage ratio; IHC. Immunohistochemistry; Aggrecan. Aggregating proteoglycan; Collagen II. Type II collagen; MMP13. Matrix metalloproteinase 13; SEM. Standard error of the mean.

Subsequently, we investigated whether PDP2 overexpression can reverse cartilage degradation in cartilage *FTO* depletion mice (FTO cKO mice) through intra-articular injection of Ad-PDP2 viruses into the knee joint of both HFD-fed and HFD+DMM in FTO cKO background mice (**Additional file 1:** Fig. S7a**)**. The PDP2 overexpression efficiency was identified by IHC staining (**Additional file 1:** Fig. S7b). The results showed that Ad-PDP2 viruses strongly improved the cartilage condition in both HFD-fed and HFD+DMM FTO cKO mice, evidenced by reduced OARSI scores, elevated HC/CC ratios, decreased lipid deposition, and fewer TUNEL positive cells (Fig. 7**a, b**). Additionally, IHC staining also confirmed the upregulation in Aggrecan and Collagen Ⅱ levels and downregulation in MMP13 expression after PDP2 overexpression (Fig. 7**c**). Following Ad-PDP2 injection, the synovitis condition was improved in both HFD-fed and HFD+DMM FTO cKO mice (**Additional file 1:** Fig. S7c).

### 3.6 YTHDF2 mediated the m^6^A modification of FTO to PDP2

**Fig. 8:**
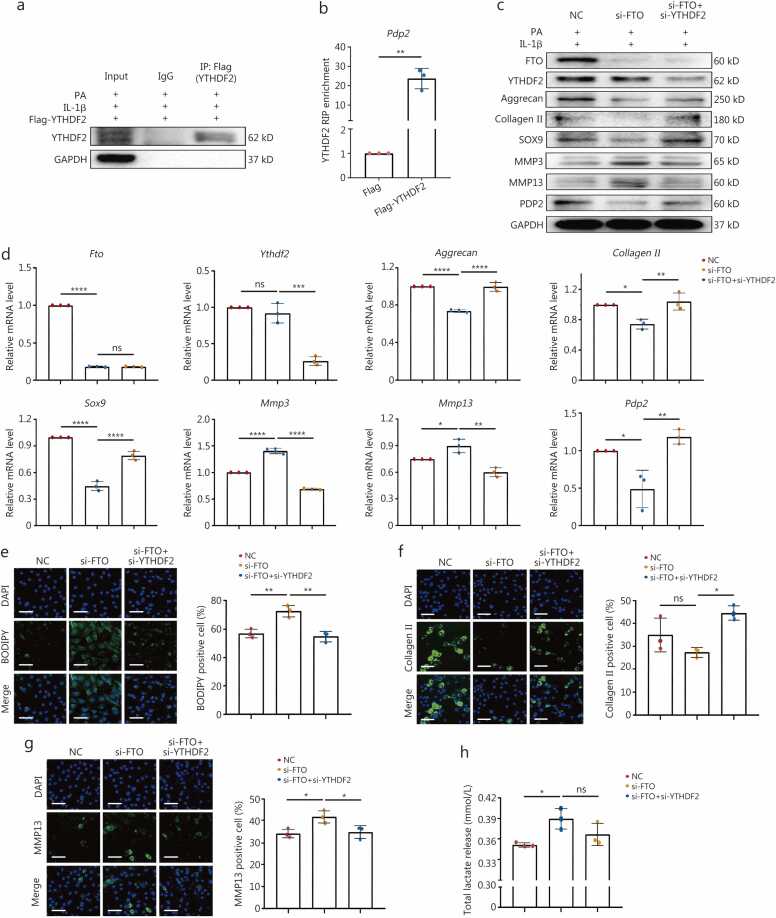
FTO modulated PDP2 expression in an m^6^A-dependent manner through YTHDF2. **a** Co-immunoprecipitation showing FTO was successfully overexpressed after Flag-YTHDF2 overexpression. **b** RIP assays showing YTHDF2 interacted with *Pdp2* mRNA. **c** FTO, YTHDF2, Aggrecan, Collagen Ⅱ, SOX9, MMP3, MMP13, and PDP2 expression after *Ythdf2* or *Fto* knockdown in chondrocytes treated with PA+IL-1β. **d***Fto, Ythdf2*, *Aggrecan*, *Collagen Ⅱ*, *Sox9*, *Mmp3*, *Mmp13*, and *Pdp2* mRNA levels after *Ythdf2* or *Fto* knockdown in chondrocytes treated with PA+IL-1β. **e** BODIPY staining of NC, si-FTO, and si-FTO+si-YTHDF2 transfected chondrocytes treated with PA+IL-1β (scale bar=200 µm). **f** Collagen Ⅱ staining of NC, si-FTO, and si-FTO+si-YTHDF2 transfected chondrocytes treated with PA+IL-1β (scale bar=200 µm). **g** MMP13 staining of NC, si-FTO, and si-FTO+si-YTHDF2 transfected chondrocytes treated with PA+IL-1β (scale bar=200 µm). **h** Lactate levels in the medium of NC, si-FTO, and si-FTO+si-YTHDF2 transfected chondrocytes treated with PA+IL-1β. The data are presented as mean±SEM. ^⁎^*P*<0.05, ^⁎⁎^*P*<0.01, ^⁎⁎⁎^*P*<0.001, ^⁎⁎⁎⁎^*P*<0.0001, ns non-significant. FTO. Fat mass and obesity associated gene; PDP2. Pyruvate dehydrogenase phosphatases2; m^6^A. N^6^-methyladenine; YTHDF2. YTH N^6^-methyladenosine RNA binding protein 2; RIP. RNA immunoprecipitation; Aggrecan. Aggregating proteoglycan; Collagen Ⅱ. Type II collagen; SOX9. Sex determining region Y-box 9; MMP3. Matrix metalloproteinase 3; MMP13. Matrix metalloproteinase 13; PA. Palmitic acid; IL-1β. Interleukin-1β; si-FTO. Small interfering RNA-FTO; si-YTHDF2. Small interfering RNA-YTHDF2; BODIPY. Boron-dipyrromethene; SEM. Standard error of the mean.

In addition to m^6^A writers and erasers, m^6^A readers also play distinct roles in regulating various inflammatory events [41]. YTHDF2, the most studied reader that mediates mRNA decay [42], is also found to be involved in inflammation. A previous study demonstrated that YTHDF2 accelerates the decay of m^6^A-modified transcripts encoding negative regulators of nuclear factor κB (NF-κB), thereby modulating NF-κB signaling in intratumoral Treg cells [43]. To investigate how m^6^A modification regulates PDP2 expression, we performed immunoprecipitation, confirming successful YTHDF2 transfection (Fig. 8**a**), RIP experiments further verified YTHDF2 interacted with *Pdp2* mRNA (Fig. 8**b**). Fig. 8**c, d** and **Additional file 1:** Fig. S7d showed the knockdown efficiency of *Ythdp2* which remarkably reversed chondrocytes degeneration caused by si-FTO as evidenced by increased Aggrecan, Collagen Ⅱ, and SOX9, decreased MMP3 and MMP13 in PA+IL-1β treated chondrocytes. *Ythdp2* knockdown also restored *PDP2* at both protein and mRNA levels, which was reduced by si-FTO and had no effect on FTO expression. BODIPY staining showed that the increase in lipid deposition induced by *Fto* knockdown was remarkably reversed by *Ythdp2* knockdown (Fig. 8**e**). Immunostaining confirmed the role of YTHDF2 in Collagen Ⅱ **(**Fig. 8**f)** and MMP13 expression in regulating si-FTO transfection (Fig. 8**g**). Notably, while si-FTO increased lactate levels, *Ythdp2* knockdown did not affect lactate production (Fig. 8**h**).

We confirmed that YTHDF2 overexpression significantly reduced Aggrecan, Collagen Ⅱ, and SOX9 levels while increasing MMP3 and MMP13 levels in FTO-overexpressed chondrocytes. Additionally, YTHDF2 overexpression decreased *PDP2* expression at both protein and mRNA levels without altering FTO expression, indicating that FTO regulates PDP2 through YTHDF2 (**Additional file 1:** Fig. S7e**, f**).

## 4 Discussion

Our study revealed that downregulated FTO in OA inhibited PDP2 expression, a central regulator of lipid metabolism, via an m^6^A-dependent mechanism, thereby promoting lipid deposition and cartilage degeneration through YTHDF2. Dysregulated FTO-guided m^6^A modifications are an important trigger of HFD-induced cartilage dysfunction. However, HFD alone induced limited cartilage loss; we thus further added IL-1β in the cell culture and performed DMM surgery on mice to better mimic the pathogenesis of OA, which is known to be a multifactorial disease [44]. Experimental results disclosed that IL-1β co-stimulation (in chondrocytes) or DMM surgery (in HFD-fed mice) accelerated lipid deposition in the cartilage, suggesting that inflammation synergizes with HFD and further influences lipid metabolism to some extent. Through intersecting analysis of MeRIP-Seq and RNA-Seq data, we ascertained that *Pdp2* is an FTO target gene. Additionally, RIP experiments further confirmed the interaction between FTO and *Pdp2* mRNA. A total of 5 GGAC sequences (m^6^A characteristic recognition sequence) were identified in the CDS region (exon 2) of PDP2. It was found that mutating the first m^6^A site interrupted the interaction between FTO and *Pdp2* mRNA and disrupted the role of PDP2 in protecting chondrocytes from lipid deposition and degeneration.

FTO was decreased in both PA and PA+IL-1β-treated chondrocytes, a trend also observed in HFD-fed and HFD+DMM mice. Previous literature reported that FTO alleviates cartilage damage in OA by mediating the FTO/miR-3591-5p/protein kinase AMP-activated catalytic subunit alpha 2 (PRKAA2) axis in a DMM-only model [25], consistent with our findings regarding the role of FTO in regulating inflammation. Genetically, pharmacologically, or adenoviruses induced FTO inhibition can all induce severe OA symptoms, reduce the HC/CC ratio, promote lipid deposition, and increase the number of TUNEL positive chondrocytes. Moreover, we observed that PDP2 expression was downregulated in PA-treated but not in PA+IL-1β-treated chondrocytes, supporting that PDP2 is more sensitive to lipid metabolism disorders than to inflammation. Interestingly, both FTO and PDP2 expression were found to be negatively correlated with BMI, providing a better understanding of their roles in regulating weight and inflammation in OA pathogenesis.

Multiple isoforms have been found to contribute to the complexity of PDH regulation by PDK and PDP, including four PDK (PDK1−4) and two PDP (PDP1 and 2) [45]. Changes in insulin-stimulated PDH phosphorylation are positively correlated with changes in plasma lactate in response to insulin or PDP1, PDP2, PDK2, and PDK4. In an earlier study, a 12-week endurance- or strength-oriented exercise training program was reported to improve insulin-stimulated PDH dephosphorylation, which was associated with a reduced lactate response [46]. PDP2 can positively regulate PDH activity and has been reported to modulate FA oxidation. For example, in mitochondrial calcium uniporter (MCU^-/-^) myofibers, PDP2 overexpression drastically reduced FA dependency [47]. Our results showed that *Pdp2* knockout aggravated the HFD-induced cartilage loss, indicating that PDP2 plays a vital role in the cartilage metabolism of OA. In HFD+DMM mice, although PDP2 expression was not significantly reduced compared to HFD-fed mice, *Pdp2* knockout still effectively modulated OA severity, and its overexpression reversed cartilage degeneration in FTO cKO mice. These findings highlight that improvement in lipid metabolism can mitigate inflammation-induced cartilage damage. Surprisingly, YTHDF2 overexpression did not affect the lactate level, providing evidence for the involvement of other unknown cellular mechanisms underlying FTO’s function; targets other than PDP2 may also account for lactate production. It is noteworthy that PPARγ may be involved in the dysregulation of the FTO-PDP2 axis.

Mechanistically, m^6^A exerts its effects primarily by recruiting m^6^A-binding proteins [48]. YTHDF2, a reader protein, displays a 10- to 50-fold higher affinity for methylated mRNAs than for nonmethylated mRNAs [49], [50]. YTHDF2 selectively binds m^6^A sites and mediates the well-documented instability of m^6^A-containing mRNAs [51]. In our study, *Fto* knockout reduced the stability of *Pdp2* mRNA, suggesting that the m^6^A reader YTHDF2 may be involved in this process. We further investigated whether YTHDF2 affects the *Pdp2* mRNA level via m^6^A regulation and found that *Ythdp2* knockdown decreased the expression and stability of PDP2 and promoted cartilage degeneration, and vice versa. Unexpectedly, the off-target role of FB23 on DHODH was identified in our study, indicating it’s not an ideal FTO inhibitor. We also can’t rule out the possible off-target effects of siRNAs and shRNAs used in our study; however, the multiple inhibiting or knockdown approaches in our study still support FTO’s role in OA. The utilization of cartilage-specific *Fto* knockout mice, which should be the most specific way of *Fto* knockout in chondrocytes, further supported our conclusions.

Collectively, we identified an FTO-dependent m^6^A demethylation modulation that inhibits PDP2 expression in HFD- and HFD+DMM-induced OA progression, acting at least partially through YTHDF2-mediated mRNA decay. Given the functional importance of the FTO/PDP2 axis in OA pathogenesis, targeting the FTO-m^6^A-PDP2 axis may represent a promising therapeutic strategy for treating both HFD-induced and inflammation-dominant forms of OA.

## 5 Conclusions

Our study identifies that downregulation of FTO exerts a pivotal effect on OA with obesity; FTO drives disease progression by regulating PDP2 activity, and YTHDF2 mediates the m^6^A modification of FTO to PDP2. Targeting the FTO/YTHDF2/PDP2 axis offers promising therapeutic potential for OA treatment.

## Abbreviations

**Table:** 

DAPI	4',6-diamidino-2-phenylindole
Acss	Acetyl-CoA synthetase
Acaca	Acetyl-coenzyme A carboxylase alpha
Acsl1	Acyl-CoA synthetase 1
Acot	Acyl-CoA thioesterases
Ad	Adenovirus
Adamts4	A disintegrin and metalloproteinase with thrombospondin motifs-4
Aggrecan	Aggregating proteoglycan
ALKBH5	AlkB homolog 5
ant	Antimycin A
BMI	Body mass index
BODIPY	Boron-dipyrromethene
FCCP	Carbonylcyanide-p-trifluoromethoxyphenylhydrazone
CDS	Coding sequence
Ctrl	Control
DAPK1	Death-associated protein kinase 1
DEGs	Differentially expressed genes
DMM	Destabilized medial meniscus
DHODH	Dihydroorotate dehydrogenase
ELISA	Enzyme-linked immunosorbent assay
Fasn	Fatty acid synthase
FTO	Fat mass and obesity associated gene
GAPDH	Glyceraldehyde-3-phosphate dehydrogenase
GO	Gene Ontology
HFD	High fat diet
IHC	Immunohistochemistry
HC/CC	Hyaline cartilage-to-calcified cartilage
KEGG	Kyoto Encyclopedia of Genes and Genomes
LDH	Lactate dehydrogenase
IL-1β	Interleukin-1β
MMPs	Matrix metalloproteinases
MeRIP	Methylated RNA immune-precipitation
METTL	Methyltransferase-like
m^6^A	N^6^-methyladenine
NC	Negative control
NCD	Normal chow diet
NF-κB	Nuclear factor κB
OA	Osteoarthritis
OARSI	Osteoarthritis Research Association
OCR	Oxygen consumption rate
PA	Palmitic acid
PPARγ	Peroxisome proliferator-activated receptor γ
PDH	Pyruvate dehydrogenase
PDP2	Pyruvate dehydrogenase phosphatases2
Pdk	Pyruvate kinase
PRKAA2	Protein kinase AMP-activated catalytic subunit alpha 2
qPCR	Quantitative reverse transcription polymerase chain reaction
RBM15	RNA-binding motif protein 15
Rot	Rotenone
SOX9	Sex determining region Y-box 9
Slc30a1	Solute carrier family 30 member 1
Slc38a1	Solute carrier family 38 member 1
SEM	Standard error of the mean
Scd	Stearoyl-CoA desaturase
SMAD2	Suppressor of variegation, enhancer of zeste, trithorax and myeloid nervy DEAF-1 domain-containing protein 2
TUNEL	Terminal deoxynucleotidyl transferase dUTP nick end labeling
Collagen II	Type II collagen
Veh	Vehicle
VIRMA	Vir like N^6^-methyladenosine (m^6^A) methyltransferase associated protein
WTAP	Wilms tumor 1-associated protein
YTHDF2	YTH N^6^-methyladenosine RNA binding protein 2
ZC3H13	Zinc-finger CCCH-type containing 13

## Ethics approval and consent to participate

The clinical ethics of this study have been approved by the Ethics Committee of Tongji Hospital, Tongji Medical College, Huazhong University of Science and Technology (TJ-IRB202503074). The animal ethics of this study have been approved by the Ethics Committee on Animal Experimentation of the Tongji Hospital (TJH-202209026).

## Funding

This work was supported by the National Natural Science Foundation of China (82472495 to Zhen-Han Deng, 82472559 to Chun-Wu Zhang), the Medical and Health Research Project of Zhejiang Province (2024KY1270 to Sheng-Wu Yang), the Key Discipline of Traditional Chinese Medicine of Zhejiang Province-Clinical Integration of Chinese and Western Medicine (TCM Orthopedics and Traumatology) of the First Affiliated Hospital of Wenzhou Medical University (2024-XK-50 to Chun-Wu Zhang), Zhejiang Clinovation Pride (Fragility Fracture Primary Osteoporosis) (to Chun-Wu Zhang), and the Provincial Advantageous Characteristic Discipline of Wenzhou Medical University (Clinical Medicine) (to Zhen-Han Deng).

## Data Availability

The data supporting this study’s findings are available from the corresponding author upon reasonable request.

## Footnotes

Appendix A Supplementary data associated with this article can be found in the online version at doi:10.1016/j.mmr.2026.100054.
