# Supplementary material for: Downregulation of FTO aggravates osteoarthritis with obesity by erasing m6A methylation of PDP2
Source: Mil Med Res. 2026 Jul 17;13(1):100054. doi: 10.1016/j.mmr.2026.100054 (PMC13400393; doi:10.1016/j.mmr.2026.100054)
Supplement: Supplementary file 1 — Supplementary material [file mmc1.pdf]

Methods

Reagents and antibodies

Recombinant mouse interleukin-1β (IL-1β) was purchased from R&D Systems (401-ML-005/CF, Minneapolis, MN, USA). Lipofectamine 3000 reagent was obtained from Invitrogen (L3000008, Waltham, MA, USA). Anti-P65 (8242), phosphorylated-P65 (p-P65, 3033), and anti-peroxisome proliferator-activated receptor γ (PPARγ, 2435T) antibodies were supplied by Cell Signaling Technology (Beverly, MA, USA). Collagen II antibody was purchased from Santa Cruz (sc-52658, Dallas, Texas, USA). Aggrecan (13880-1-AP), matrix metalloproteinase (MMP) 3 (66338-1-Ig), MMP13 (18165-1-AP), fat mass and obesity associated gene (FTO) (27226-1-AP), and YTH N^6-methyladenosine RNA binding protein 2 (YTHDF2) (24744-1-AP) antibodies were obtained from Proteintech (Wuhan, Hubei, China). Sex determining region Y-box 9 (SOX9) (A2479), Flag-Tag (AE092), and pyruvate dehydrogenase phosphatases 2 (PDP2) (A17190) antibodies were purchased from Abclonal (Wuhan, Hubei, China). GAPDH (BM3874) antibody was purchased from Boster (Wuhan, Hubei, China), and secondary antibodies were obtained from The Jackson Laboratory (New York, USA). Boron-dipyrromethene (BODIPY) was from GLPBIO (Montclair, CA, USA). FTO inhibitor FB23 (HY-137187) and dihydroorotate dehydrogenase (DHODH) inhibitor Brequinar (Breq, HY-108325) were from MedChemExpress (Shanghai, China). Cell counting kit-8 (CCK-8) kits were obtained from Boster Biotechnology (Wuhan, Hubei, China).

Cell viability assay

Chondrocytes were plated on 96-well plates and incubated with control (Ctrl) or 200 μmol/L palmitic acid (PA), or 200 μmol/L PA and 5 ng/ml IL-1β for 24 h. Cell viability was assayed using a CCK-8 kit according to the provided instructions. After the cells were incubated with the CCK-8 reagent for 2 h, the absorbance at 450 nm was measured using a microplate reader.

RNA sequencing

Whole-transcriptome sequencing was conducted on primary mouse chondrocytes with Ctrl, 200 μmol/L PA, or 200 μmol/L PA and 5 ng/ml IL-1β incubation for 24 h with three independent replicates per group. A total of 1000 ng of total RNA from each sample was utilized for sequencing. Paired-end 2×100 bp RNA sequencing (RNA-Seq) (Illumina TruSeq RNA Library Prep Kit, Illumina Novaseq6000, USA) was performed. The quality of raw reads was assessed using MultiQC [1].

Differential expression analysis was carried out with the R package "DEseq2". Genes with an adjusted *P*-value of <0.05 (calculated using the moderated *t*-statistic with the Benjamini-Hochberg (BH) method to control the false discovery rate [2]). Enrichment analysis of differentially expressed genes (DEGs) was performed using the R package "clusterProfiler", identifying significant Gene Ontology (GO) and Kyoto Encyclopedia of Genes and Genomes (KEGG) terms as those with a BH-adjusted *P*-value <0.05.

RNA immunoprecipitation-qPCR (RIP-qPCR)

Flag-FTO or Flag-YTHDF2 overexpressed chondrocyte pellets were lysed in lysis buffer of 150 mmol/L KCl, 10 mmol/L HEPES, 2 mmol/L EDTA, 0.5% NP-40, 0.5 mmol/L dithiothreitol (DTT), 1× protease inhibitor cocktail, and RNasin plus RNase inhibitor for 30 min at 4 °C. The lysates were centrifuged, and the supernatant was transferred to pass through a 0.45 μm membrane syringe filter. A small aliquot of lysate was saved as input, and the remaining sample was incubated with anti-Flag M2 magnetic beads (Sigma-Aldrich, Saint Louis, MO, USA) for 4 h at 4 °C. Then, the beads were eluted in wash buffer containing 0.1% SDS and 10 ml proteinase K (Invitrogen, Waltham, MA, USA) and incubated at 55 °C for 30 min. The input and immunoprecipitated RNAs were isolated using TRIzol reagent and were reverse transcribed into cDNA. The fold enrichment was detected by qPCR primers used for *Pdp2*. Site 1, 5'-TACCGAACATCATCCCATTC-3' (sense), 5'-GAGTACCAAATTCAGTGTTAAGG-3' (antisense). Site 2, 5'-ATCCTGCGTTGGCTCAA-3' (sense), 5'-AATGCTCAGCCCCATTTC-3' (antisense). Site 3, 5'-ACATGCTGGGGAATGAGGA-3' (sense), 5'-AGCAGGCTCTGCATGA-3' (antisense). Site 4, 5'-AGTCCGAGGACAGGACA-3' (sense), 5'-AGATGTTGAGGGCCTC-3' (antisense).

RNA extraction and RT-qPCR

Total RNA was extracted from cultured chondrocytes using TRIzol reagent according to the manufacturer's instructions. A total of 1 μg RNA was used to synthesize cDNA utilizing Revert Aid First Strand cDNA synthesis Kit (11300ES, Yeason, Shanghai, China). Templates were amplified using the SYBR Green Quantitative PCR Protocol (Q221-01, Yeason, Shanghai, China) to determine mRNA levels. All primer sequences used are listed below: *Fto*, 5'-GAGGATGAAAGTGAGGACG-3' (sense), 5'-TGGCATTGAGGTCATCCA-3' (antisense); *Pdp2*, 5'-GGACGAGGATACGAGGCTGA-3' (sense), 5'-GCGTCTCCCACCTCGTAAAA-3' (antisense); *Ythdf2*, 5'-GAGCAGAGACCAAAAGGTCAAG-3' (sense), 5'-CTGTGGGCTCAAGTAAGGTTC-3'

(antisense); *Gapdh*, 5’-AGGTCGGTGTGAACGGATTTG-3’ (sense), 5’-TGTAGACCATGTAGTTGAGGTCA-3’ (antisense); *Aggrecan*, 5’-CCTGCTACTTCATCGACCCC-3’ (sense), 5’-AGATGCTGTTGACTCGAACCT-3’ (antisense); *Collagen II*, 5’-GGGAATGTCCTCTGCGATGAC-3’ (sense), 5’-GAAGGGGATCTCGGGGTTG-3’ (antisense); *Sox9*, 5’-GAGCCGGATCTGAAGAGGGA-3’ (sense), 5’-GCTTGACGTGTGGCTTGTTC-3’ (antisense); *Mmp3*, 5’-ACATGGAGACTTTGTCCCTTTTG-3’ (sense), 5’-TTGGCTGAGTGGTAGAGTCCC-3’ (antisense); *Mmp13*, 5’-CTTCTTCTTGTTGAGCTGGACTC-3’ (sense), 5’-CTGTGGAGGTCACTGTAGACT-3’ (antisense). The relative mRNA levels of target genes were calculated using the $2^{-\Delta\Delta Ct}$ method.

Western blotting analysis

Cultured chondrocytes were lysed by ice-cold RIPA lysis buffer (Boster, Wuhan, China) containing 1% protease and phosphatase inhibitors for 30 min. Then, the cell lysates were immediately centrifuged at 10,000 *g* at 4 °C for 30 min. Equal amounts of protein were loaded on SDS-PAGE (10%−15%) for electrophoresis, and then the proteins were transferred to polyvinylidene fluoride (PVDF) membranes. Each membrane was blocked with 5% non-fat milk in Tris-buffered saline with 0.1% Tween 20 buffer (TBST) for 1 h and then incubated with primary antibodies overnight at 4 °C on a shaker. After incubation with horseradish peroxidase-conjugated secondary antibodies for 1 h, proteins were visualized by an enhanced chemiluminescence kit (Thermo Fisher Scientific, Waltham, MA, USA) in the ChemiDoc XRS System (Bio-Rad Laboratories, Hercules, CA, USA). All experiments were independently repeated three times. Proteins were quantified via Image Lab Software (Bio-Rad Laboratories, Hercules, CA, USA), and targeted protein levels were normalized to GAPDH, whereas p-P65 protein levels were normalized by total P65.

DHODH activity assay

Primary mouse chondrocytes were cultured and treated with 5 μmol/L FB23 or DHODH inhibitor Breq for 24 h. DHODH activity was measured from cell lysates using a mouse DHODH ELISA kit (SEJ271Mu, Cloud-Clone Corp, Wuhan, China), according to the manufacturer’s instructions.

Lactate concentration measurement

Primary mouse chondrocytes were cultured and transfected with si-FTO, wild-type PDP2 (PDP2-W), mutant PDP2 (PDP2-M), orp-YTHDF2, and subjected to PA and IL-1β treatment for 24 h. The lactate

concentrations from cell culture media were measured using an ELISA lactate kit (BA1698, Saintbio, Shanghai, China) according to the manufacturer's instructions.

Pyruvate dehydrogenase activity assay

Pyruvate dehydrogenase (PDH) activity was measured according to the protocol provided by the manufacturer (AKAC005C, Boxbio, Beijing, China). Chondrocytes were transfected with NC, wild-type, or mutant *PDP2* plasmids. Two days after transfection, cell medium was collected and incubated with substrates by measuring the absorbance of the complex at 605 nm. PDH activity was calculated and normalized with NC-transfected medium.

Seahorse assay

Basal oxygen consumption rate (OCR) was measured using a Seahorse XFe24 analyzer (Agilent Technologies, Santa Clara, CA, USA) according to the manufacturer's instructions. Primary chondrocytes were seeded in an XF24 culture microplate (4×10^4 cells/well) and were transfected with p-NC or p-PDP2 plasmids for 24 h; the cells were subjected to PA or PA+IL-1β incubation for another 24 h. Cells were pre-equilibrated for 1 h in the unbuffered XF assay medium supplemented with 25 mmol/L glucose and 1 mmol/L sodium pyruvate. Three or more consecutive measurements were obtained under basal conditions and after the sequential addition of compounds at the following final concentrations: 1 μmol/L oligomycin, 1.5 μmol/L carbonylcyanide-p-trifluoromethoxyphenylhydrazone (FCCP), and 0.5 μmol/L rotenone (Rot) and antimycin A (ant, all from Sigma-Aldrich, St. Louis, MO, USA). In this assay, basal oxygen consumption can be determined by measuring OCR in the absence of drugs, whereas maximal oxygen consumption was calculated between the injection of FCCP and Rot+ant. The readings of OCR of each well were normalized to protein amount. XFe Wave software (Agilent Technologies, Santa Clara, CA, USA) was used to analyze the results.

Table S1 Human sample information in OA patients for analyzing the expression of FTO and PDP2 correlation with BMI (*n*=33)

Number	Age (years)	Gender	BMI (kg/m^2)	Diagnose
OA1	62	Female	30.4	OA
OA2	77	Female	27.7	OA
OA3	76	Male	26.8	OA
OA4	61	Female	26.4	OA
OA5	65	Female	25.1	OA
OA6	69	Female	27.4	OA
OA7	77	Male	26.1	OA
OA8	68	Female	37.1	OA
OA9	72	Female	29.9	OA
OA10	86	Female	30.0	OA
OA11	75	Female	37.0	OA
OA12	74	Female	28.1	OA
OA13	60	Female	30.0	OA
OA14	77	Male	26.0	OA
OA15	65	Female	25.0	OA
OA16	62	Female	23.4	OA
OA17	70	Female	21.3	OA
OA18	64	Male	25.0	OA
OA19	73	Female	17.5	OA
OA20	68	Female	32.3	OA
OA21	65	Female	29.2	OA
OA22	64	Female	31.6	OA
OA23	77	Female	28.2	OA
OA24	59	Male	33.7	OA
OA25	61	Female	24.3	OA
OA26	64	Male	22.3	OA
OA27	69	Female	32.2	OA
OA28	63	Female	28.8	OA
OA29	74	Female	18.3	OA
OA30	67	Female	21.4	OA
OA31	72	Male	24.5	OA
OA32	69	Female	31.2	OA
OA33	75	Male	20.4	OA

OA. Osteoarthritis; FTO. Fat mass and obesity associated gene; PDP2. Pyruvate dehydrogenase phosphatases 2; BMI. Body mass index

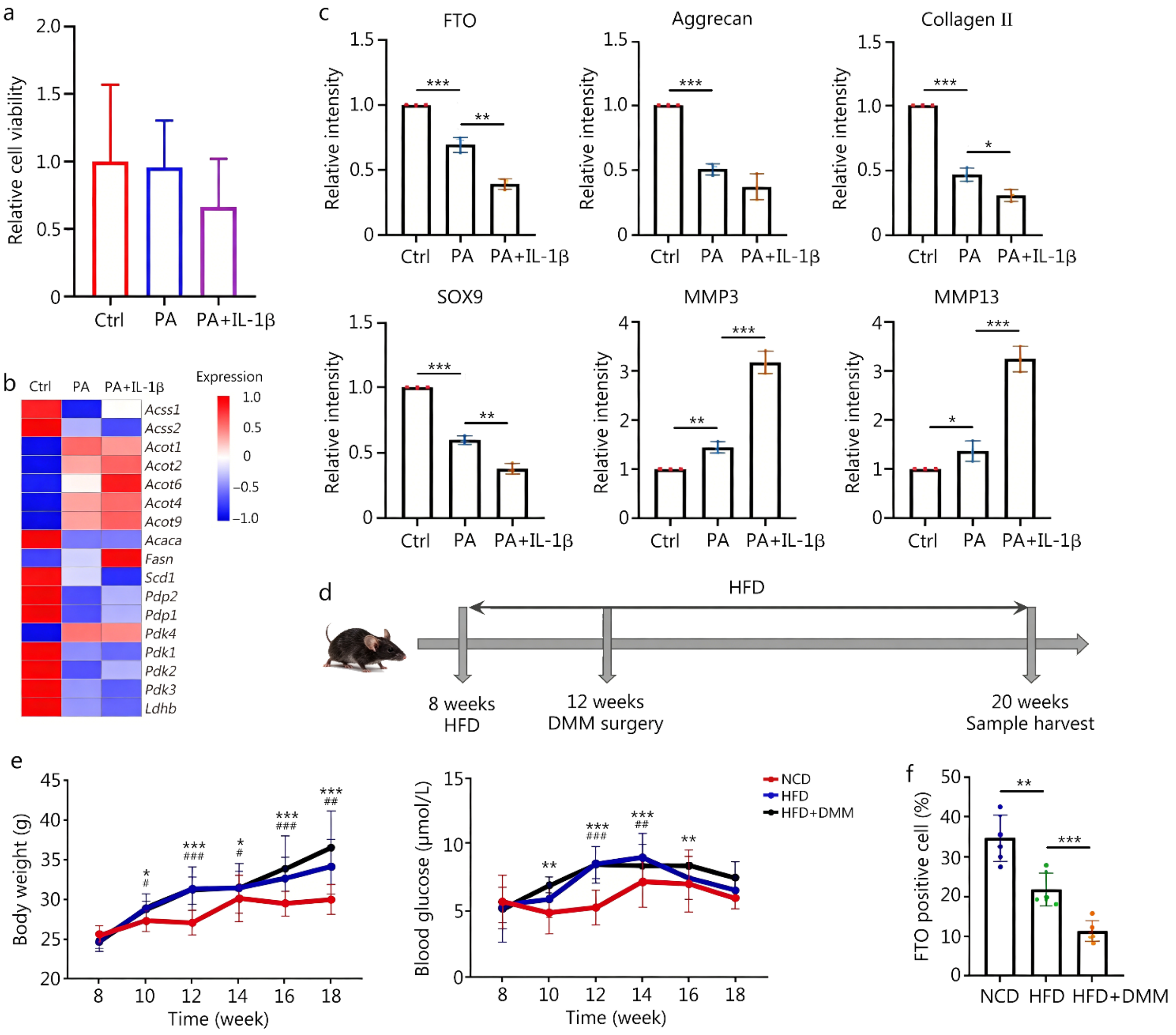

Fig. S1 Reduced FTO expression in human OA samples, HFD and HFD+DMM animal models of OA, and *in vitro* mouse chondrocytes. **a** Cell viability of chondrocytes treated with Ctrl, 200 μmol/L PA, or PA+5 ng/ml IL-1β for 24 h. **b** RNA sequencing analysis revealed changes in lipid metabolism genes in chondrocytes stimulated with Ctrl, 200 μmol/L PA, or PA+5 ng/ml IL-1β for 24 h. **c** Quantification of Western blotting results in **Fig. 1e**. **d** Schematic illustration of HFD and HFD+DMM model design for C57BL/6J mice. **e** Body weight and blood glucose levels of NCD, HFD, and HFD+DMM mice following experiments. HFD vs. NCD, *P<0.05, **P<0.01, ***P<0.001; HFD+DMM vs. NCD, $^{\#}P$<0.05, $^{\#\#}P$<0.01, $^{\#\#\#}P$<0.001. **f** Quantification of FTO staining in **Fig. 1o**. OA. Osteoarthritis; Ctrl. Control; PA. Palmitic acid; IL-1β. Interleukin-1β; Acss1. Acetyl-CoA synthetase 1; Acss2. Acetyl-CoA synthetase; Acot1. Acyl-CoA thioesterases 1; Acot2. Acyl-CoA thioesterases 2; Acot6. Acyl-CoA thioesterases 6; Acot4. Acyl-CoA thioesterases 4; Acot9. Acyl-CoA thioesterases 9; Acaca. Acetyl-coenzyme A carboxylase alpha; Fasn. Fatty acid synthase; Scd1. Stearoyl-CoA desaturase 1; sh. Short hairpin1; Pdp2. Pyruvate dehydrogenase phosphatases 2; Pdp1. Pyruvate dehydrogenase phosphatases1; Pdk4. Pyruvate kinase4; Pdk1. Pyruvate kinase 1;

Pdk2. Pyruvate kinase 2; Pdk3. Pyruvate kinase 3; Ldhb. Lactate dehydrogenase B. FTO. Fat mass and obesity associated gene; Aggrecan. Aggregating proteoglycan; Collagen II. Type II collagen; SOX9. Sex determining region Y-box 9; MMP3. Matrix metalloproteinase 3; MMP13. Matrix metalloproteinase 13; NCD. Normal chow diet; HFD. High fat diet; DMM. Destabilized medial meniscus

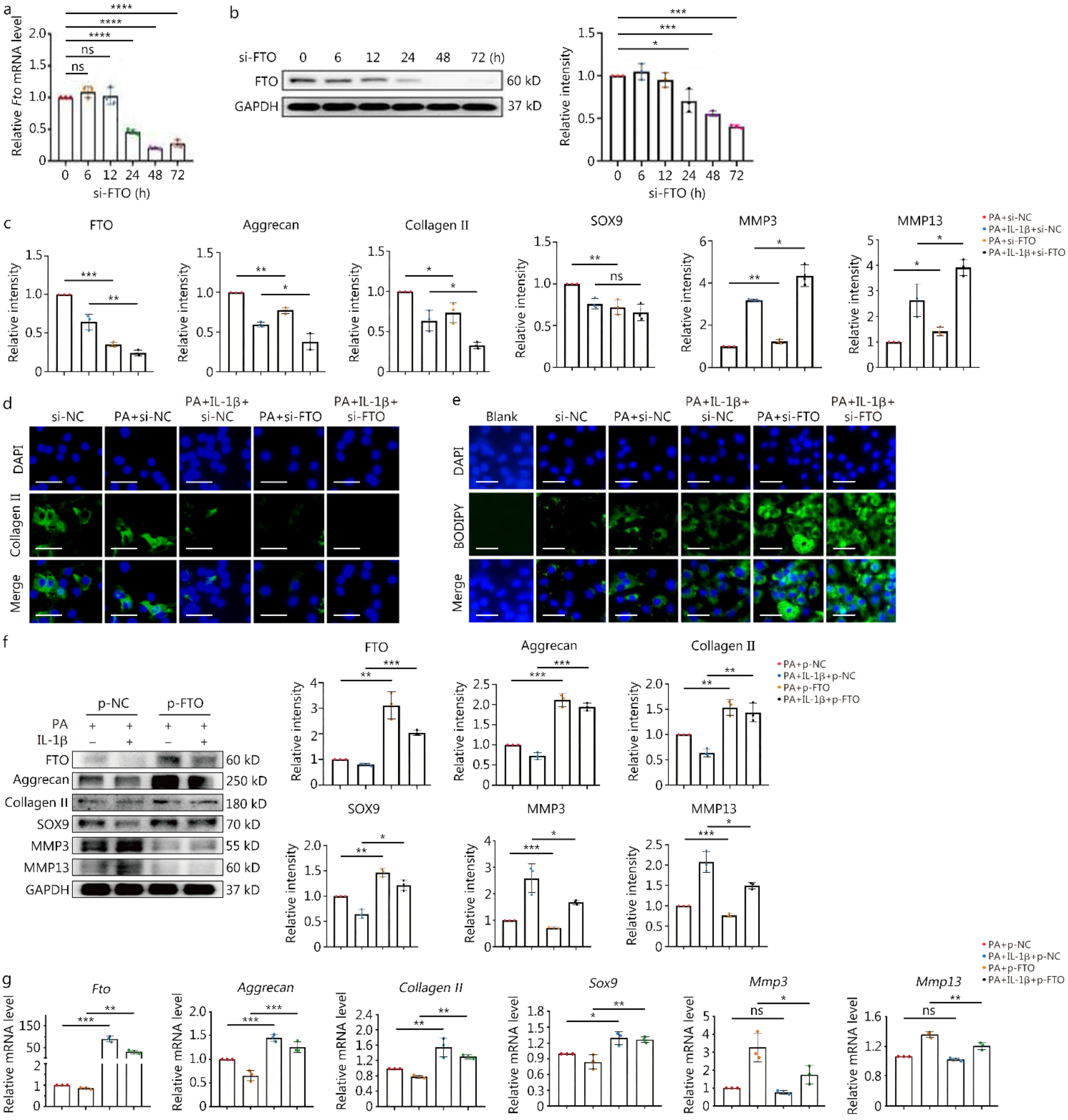

Fig. S2 FTO modulates cartilage degeneration and inflammation. **a** *Fto* mRNA levels after chondrocytes were transfected with si-FTO for 0, 6, 12, 24, 48, 72 h. **b** FTO protein levels after chondrocytes were transfected with si-FTO for 0, 6, 12, 24, 48, 72 h. **c** Quantification of Western blotting results in **Fig. 2a**. **d** Collagen II staining in chondrocytes treated with 200 μmol/L PA or PA+5 ng/ml IL-1β for 24 h with or without *Fto* knockdown (scale bar=20 μm). **e** BODIPY positive chondrocytes with or without *Fto* knockdown using si-FTO in chondrocytes treated with PA and PA+IL-1β (scale bar=20 μm). **f** Representative Western blotting showing FTO, Aggrecan, Collagen II, SOX9, MMP3, and MMP13 expression after treatment with PA and PA+IL-1β in primary mouse chondrocytes with or without FTO overexpression. **g** *Fto*, *Aggrecan*, *Collagen II*, *Sox9*, *Mmp3*, and *Mmp13* mRNA levels after treatment with PA and PA+IL-1β in primary mouse chondrocytes with or without FTO overexpression. The data are presented

as mean±SEM. $^{*}P<0.05$, $^{**}P<0.01$, $^{***}P<0.001$, ns non-significant. FTO. Fat mass and obesity associated gene; si-NC. Small interfering RNA-negative control; si-FTO. Small interfering RNA-FTO; Aggrecan. Aggregating proteoglycan; Collagen II. Type II collagen; SOX9. Sex determining region Y-box 9; MMP3. Matrix metalloproteinase 3; MMP13. Matrix metalloproteinase 13; GAPDH. Glyceraldehyde-3-phosphate dehydrogenase; PA. Palmitic acid; IL-1β. Interleukin-1β; BODIPY. Boron-dipyrromethene; DAPI. 4',6-diamidino-2-phenylindole; p-P65. Phosphorylated-P65; SEM. Standard error of the mean

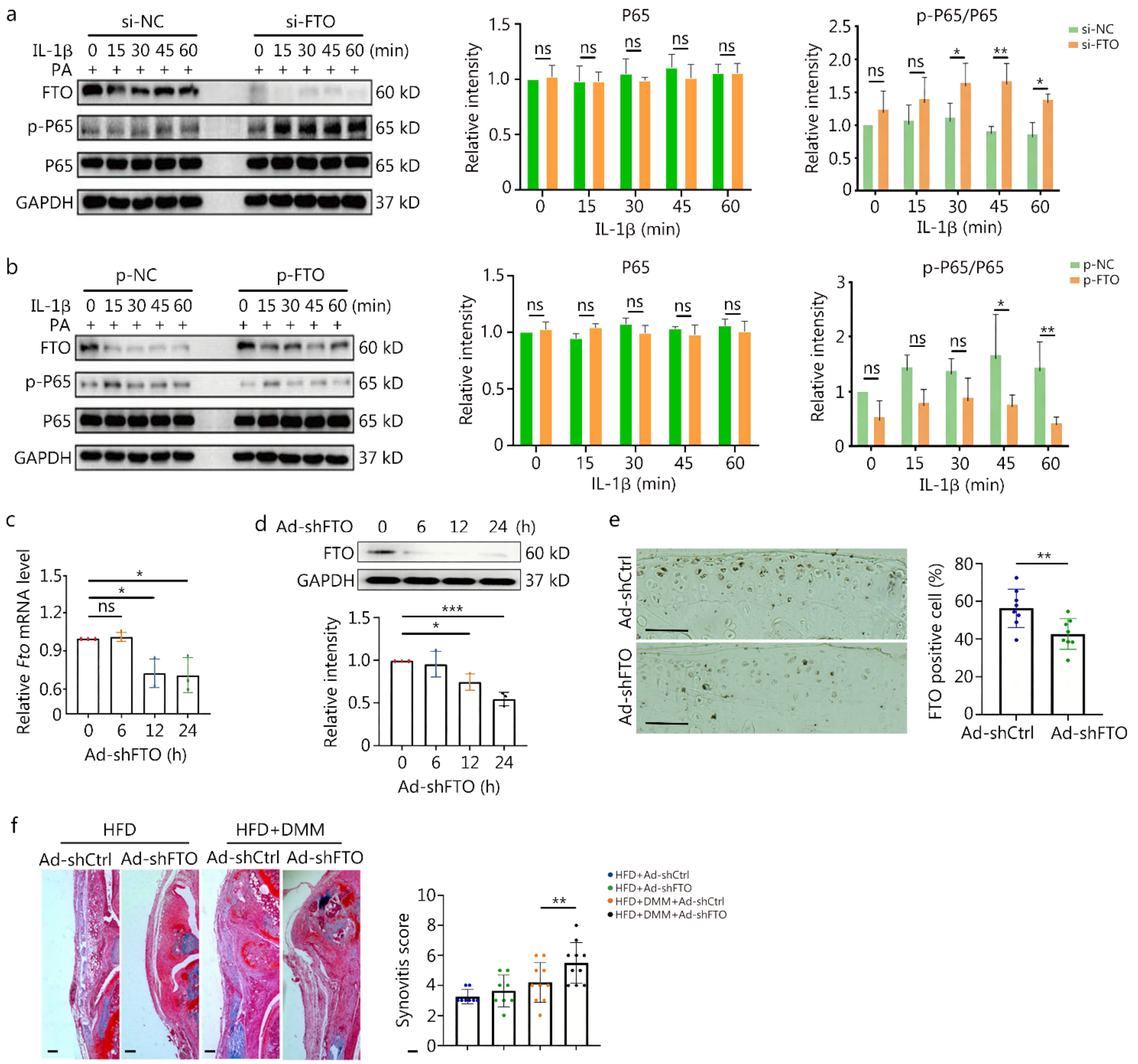

Fig. S3 FTO modulates OA pathogenesis both *in vivo* and *in vitro*. **a** Representative Western blotting of FTO, p-P65, and P65 in mouse *Fto* knockdown chondrocytes treated with PA for 24 h and following IL-1β exposure for 0, 15, 30, 45, 60 min, and quantitative analysis of P65 and the ratio of p-P65/P65. **b** Representative Western blotting of FTO, p-P65, and P65 in mouse FTO overexpression chondrocytes treated with PA for 24 h and following IL-1β exposure for 0, 15, 30, 45, 60 min, and quantitative analysis of P65 and the ratio of p-P65/P65. **c** *Fto* mRNA levels after chondrocytes were transfected with 100 MOI Ad-shFTO viruses for 0, 6, 12, 24 h. **d** FTO protein levels after chondrocytes were transfected with 100 MOI Ad-shFTO viruses for 0, 6, 12, 24 h. **e** IHC staining of FTO mice injected with Ad-Ctrl or Ad-FTO viruses (scale bar=200 μm) (*n*=6). **f** Safranin O staining for synovium tissue of mice and synovitis score in HFD and HFD+DMM mice injected with Ad-shCtrl or Ad-shFTO viruses (scale bar=200 μm) (*n*=8−10). The data are presented as mean±SEM. $^{*}P$<0.05, $^{**}P$<0.01, $^{***}P$<0.001, ns non-significant. FTO. Fat mass and obesity associated gene; OA. Osteoarthritis; p-NC. Plasmid negative control; p-FTO. Plasmid-FTO

overexpression; PA. Palmitic acid; IL-1β. Interleukin-1β; Aggrecan. Aggregating proteoglycan; Collagen II. Type II collagen; SOX9. Sex determining region Y-box 9; MMP3. Matrix metalloproteinase 3; MMP13. Matrix metalloproteinase 13; GAPDH. Glyceraldehyde-3-phosphate dehydrogenase; MOI. Multiplicity of infection; IHC. Immunohistochemistry; HFD. High-fat diet; DMM. Destabilized medial meniscus; Ad-shCtrl. Adenovirus-short hairpin control; Ad-shFTO. Adenovirus-short hairpin FTO; SEM. Standard error of the mean

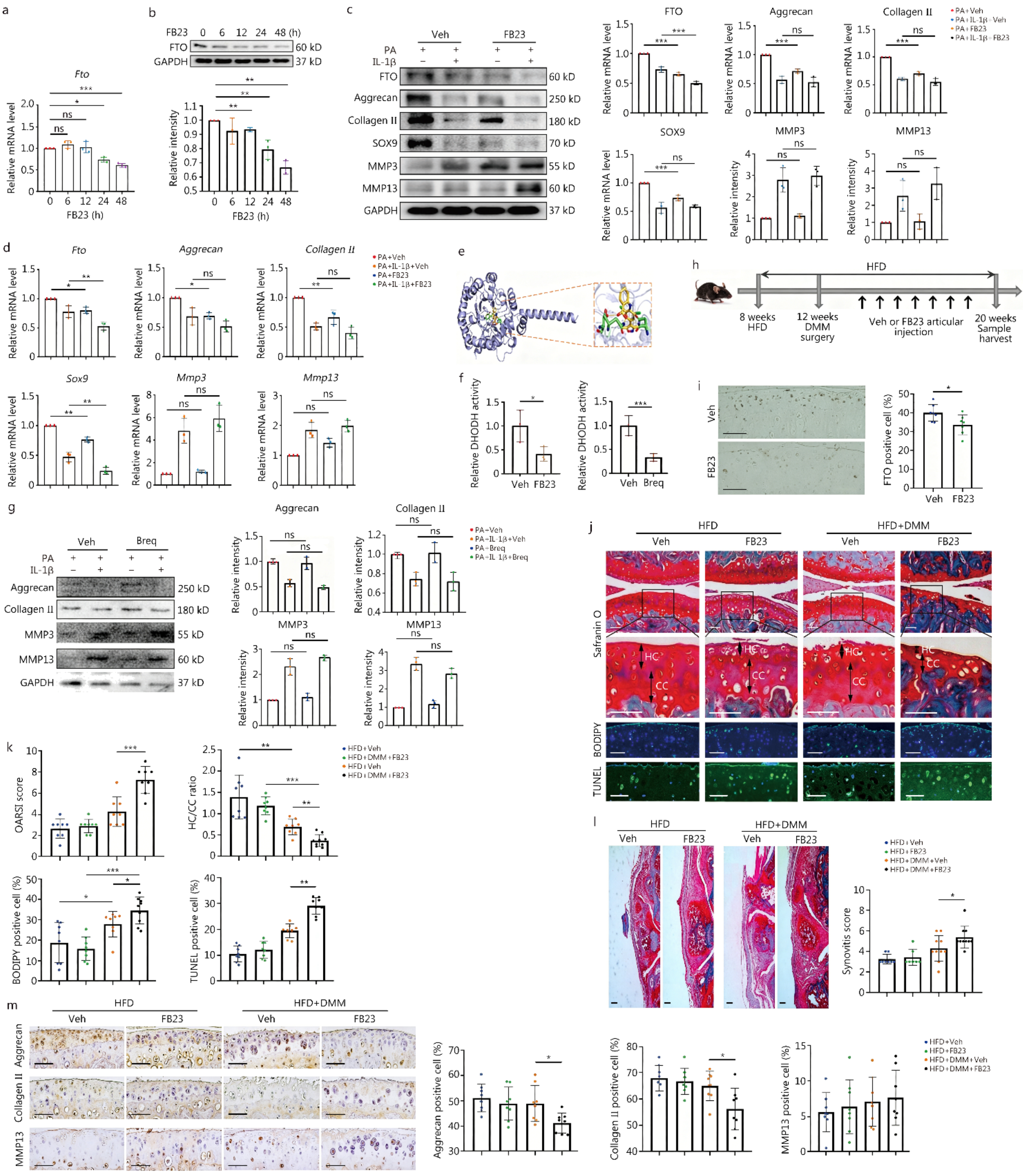

Fig. S4 The role of pharmacological blockade of FTO in OA pathogenesis. *Fto* mRNA **(a)** and protein quantification levels (**b**) after chondrocytes were treated with 5 µmol/L FB23 for 0, 6, 12, 24, 48 h. **c** Representative Western blotting showing FTO, Aggrecan, Collagen II, SOX9, MMP3, and MMP13 expression after 5 µmol/L FB23 exposure for 24 h in chondrocytes treated with PA or PA+IL-1β. **d** *Fto*, *Aggrecan*, *Collagen II*, *SOX9*, *Mmp3*, and *Mmp13* mRNA levels after 5 µmol/L FB23 exposure for 24 h in chondrocytes treated with PA or PA+IL-1β. **e** Binding modes with the binding energy generated by ZDOCK, and key residues for interaction between FB23 and mouse DHODH. **f** DHODH activity in chondrocytes treated with or without FB23 or 5 µmol/L Breq for 24 h. **g** Aggrecan, Collagen II,

MMP3, and MMP13 expression after 5 μmol/L Breq exposure for 24 h in chondrocytes treated with PA or PA+IL-1β. **h** Schematic illustration of HFD and HFD+DMM model design for C57BL/6J mice injected with 3 mg/kg FB23 weekly. **i** IHC staining of FTO in mice injected with Veh or FB23 (scale bar=200 μm) (n=7). **j** Safranin O, BODIPY, and TUNEL staining of the knee joint in HFD and HFD+DMM mice with Veh or FB23 injected (scale bar=200 μm). H. Hyaline cartilage; C. Calcified cartilage. **k** OARIS score, HC/CC ratio, BODIPY positive cells, and TUNEL positive cells in HFD and HFD+DMM mice with Veh or FB23 injected. **l** Safranin O staining for synovium tissue and synovitis score of mice in HFD and HFD+DMM mice with Veh or FB23 injected (scale bar=200 μm) (n=8). **m** IHC staining of Aggrecan, Collagen II, and MMP13 in HFD and HFD+DMM Veh or FB23 injected mice (scale bar=200 μm). The data are presented as mean±SEM. $^{*}P<0.05$, $^{**}P<0.01$, $^{***}P<0.001$, ns non-significant. FTO. Fat mass and obesity associated gene; OA. Osteoarthritis; PA. Palmitic acid; IL-1β. Interleukin-1β; Aggrecan. Aggregating proteoglycan; Collagen II. Type II collagen; SOX9. Sex determining region Y-box 9; MMP3. Matrix metalloproteinase 3; MMP13. Matrix metalloproteinase 13; GAPDH. Glyceraldehyde-3-phosphate dehydrogenase; DHODH. Dihydroorotate dehydrogenase; Breq. Brequinar; HFD. High fat diet; DMM. Destabilized medial meniscus; Veh. Vehicle; IHC. Immunohistochemistry; BODIPY. Boron-dipyrromethene; TUNEL. Terminal deoxynucleotidyl transferase dUTP nick end labeling; OARSI. Osteoarthritis Research Association; HC/CC ratio. Hyaline cartilage-to-calcified cartilage ratio; SEM. Standard error of the mean

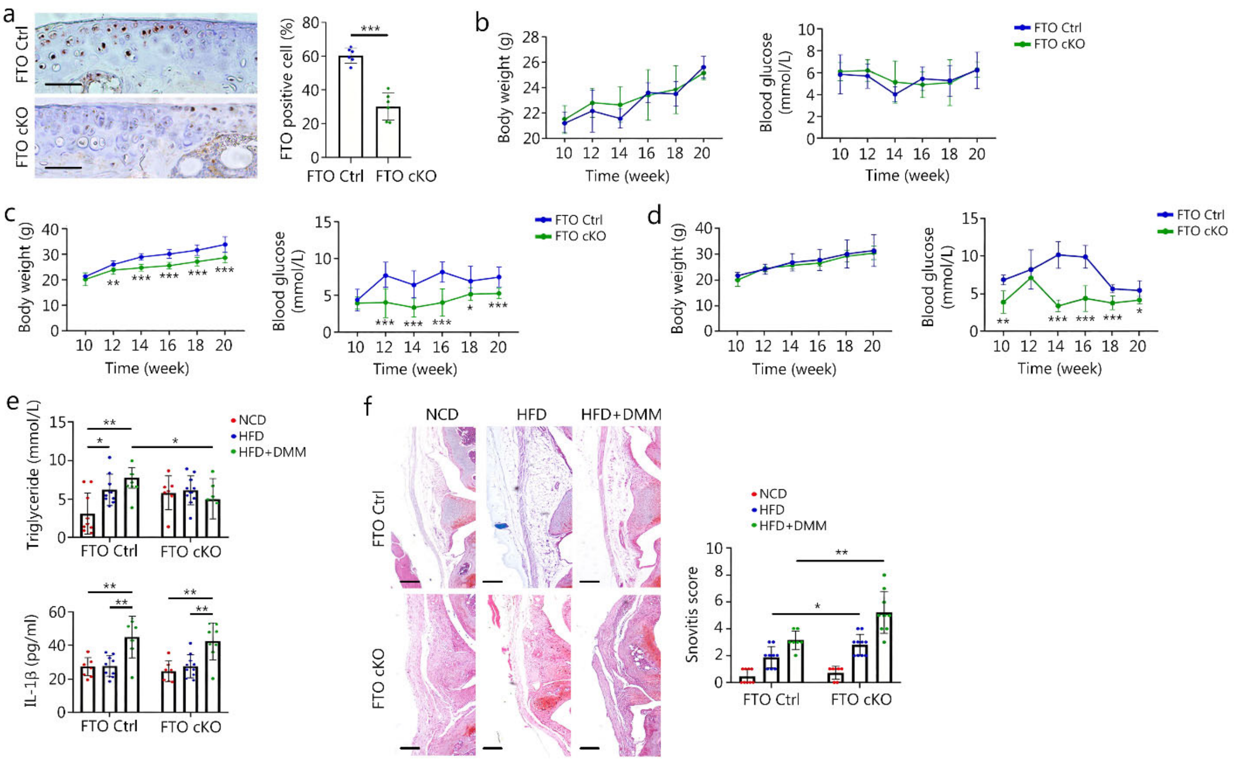

Fig. S5 Deletion of *Fto* in mouse chondrocytes promotes cartilage destruction in HFD and HFD+DMM mice. **a** IHC staining of FTO positive cells in FTO Ctrl and FTO cKO mice (scale bar=200 μm) (n=6). **b** Body weight and blood glucose levels comparison between NCD-fed FTO Ctrl and FTO cKO mice. **c** Body weight and blood glucose levels comparison between HFD-fed FTO Ctrl and FTO cKO mice. **d** Body weight and blood glucose levels comparison between HFD-fed DMM surgical FTO Ctrl and FTO cKO mice. **e** Triglyceride and IL-1β levels in NCD, HFD, and HFD+DMM FTO Ctrl and FTO cKO mice. **f** Safranin O staining of synovium and synovitis score (scale bar=200 μm) (n=7−9). The data are presented as mean±SEM. $^{*}P$<0.05, $^{**}P$<0.01, $^{***}P$<0.001, ns non-significant. FTO. Fat mass and obesity associated gene; HFD. High-fat diet; DMM. Destabilized medial meniscus; IHC. Immunohistochemistry; Aggrecan. Aggregating proteoglycan; Collagen II. Type II collagen; MMP13. Matrix metalloproteinase 13; Ctrl. Control; cKO. Conditional knockout; NCD. Normal chow diet; IL-1β. Interleukin-1β; PDP2. Pyruvate dehydrogenase phosphatases 2; SEM. Standard error of the mean

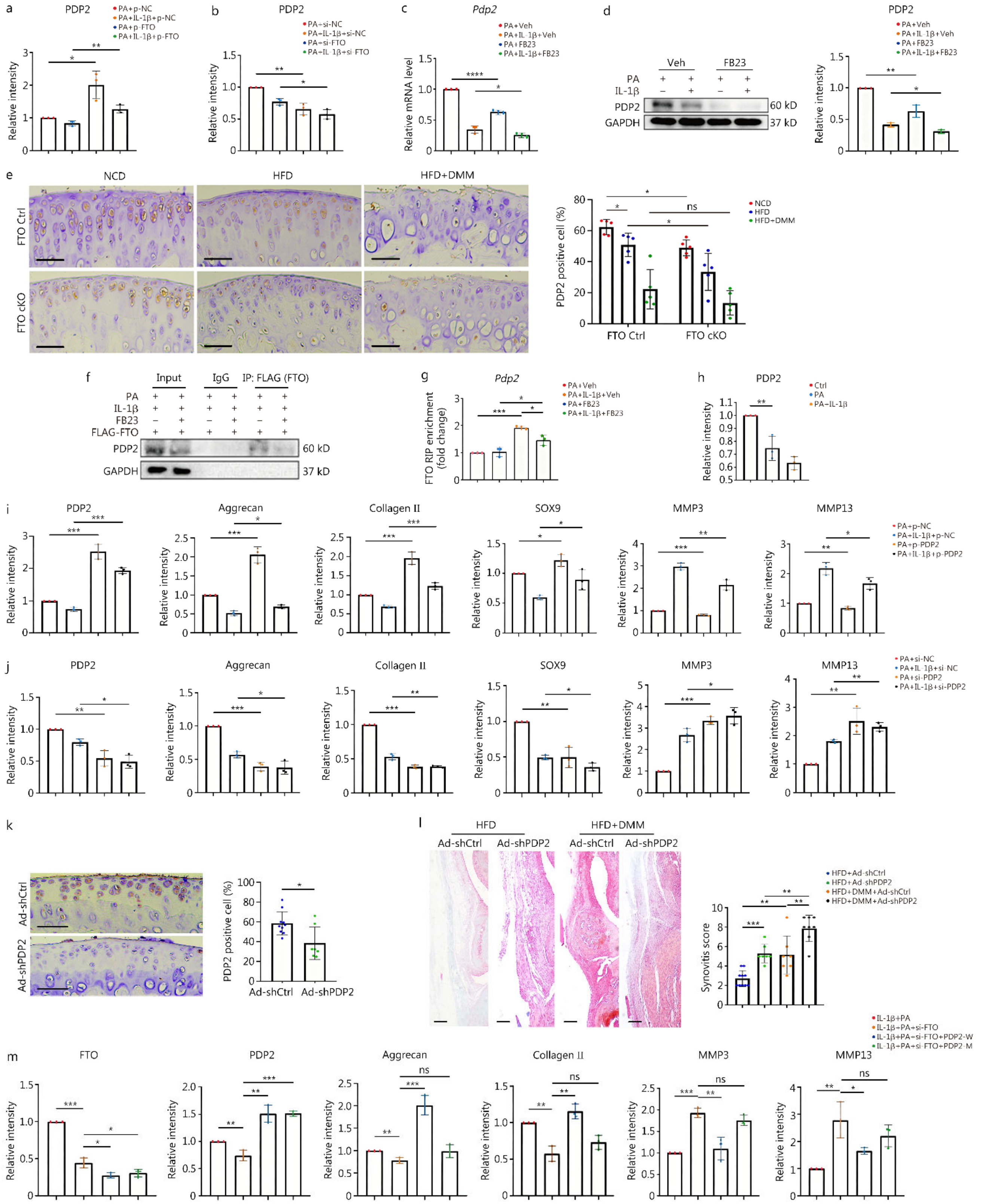

Fig. S6 PDP2 works downstream of FTO and modulates cartilage destruction in HFD and HFD+DMM mice. **a** Quantification of Western blotting results in **Fig. 4c**. **b** Quantification of Western blotting results in **Fig. 4e**. **c** *Pdp2* mRNA levels after FB23 treatment. **d** PDP2 protein levels after FB23 treatment. **e** IHC of PDP2 staining of mice in NCD, HFD, and HFD+DMM FTO Ctrl and FTO cKO mice (scale bar=200 μm) (n=7−9). **f** Western blotting showing FTO was less immunoprecipitated after FB23 incubation. **g** RIP assay indicated FTO binding with *Pdp2* mRNA after

FB23 treatment. **h** Quantification of Western blotting results in **Fig. 4i**. **i** Quantification of Western blotting results in **Fig. 5a**. **j** Quantification of Western blotting results in **Fig. 5c**. **k** IHC staining of PDP2 positive cells in Ad-shCtrl and Ad-shPDP2-injected mice (scale bar=200 μm). **l** Safranin O staining for synovium tissue and synovitis score of mice in Ad-shCtrl and Ad-shPDP2-injected HFD, HFD+DMM mice (scale bar=200 μm) (n=7−9). **m** Quantification of Western blotting results in **Fig. 6f**. The data are presented as mean±SEM. $^{*}P<0.05$, $^{**}P<0.01$, $^{***}P<0.001$, $^{****}P<0.0001$, ns non-significant. PDP2. Pyruvate dehydrogenase phosphatases2; FTO. Fat mass and obesity associated gene; PA. Palmitic acid; IL-1β. Interleukin-1β; Veh. Vehicle; si-NC. Small interfering RNA-negative control; si-PDP2. Small interfering RNA-PDP2; Aggrecan. Aggregating proteoglycan; Collagen II. Type II collagen; SOX9. Sex determining region Y-box 9; MMP3. Matrix metalloproteinase 3; MMP13. Matrix metalloproteinase 13; Ad-shCtrl. Adenovirus-short hairpin control; Ad-shPDP2. Adenovirus-short hairpin PDP2; HFD. High fat diet; DMM. Destabilized medial meniscus; IHC. Immunohistochemistry; RIP. GAPDH. Glyceraldehyde-3-phosphate dehydrogenase; RNA immunoprecipitation; PDP2-W. Wild-type pyruvate dehydrogenase phosphatases 2; PDP2-M. Mutant pyruvate dehydrogenase phosphatases 2; SEM. Standard error of the mean

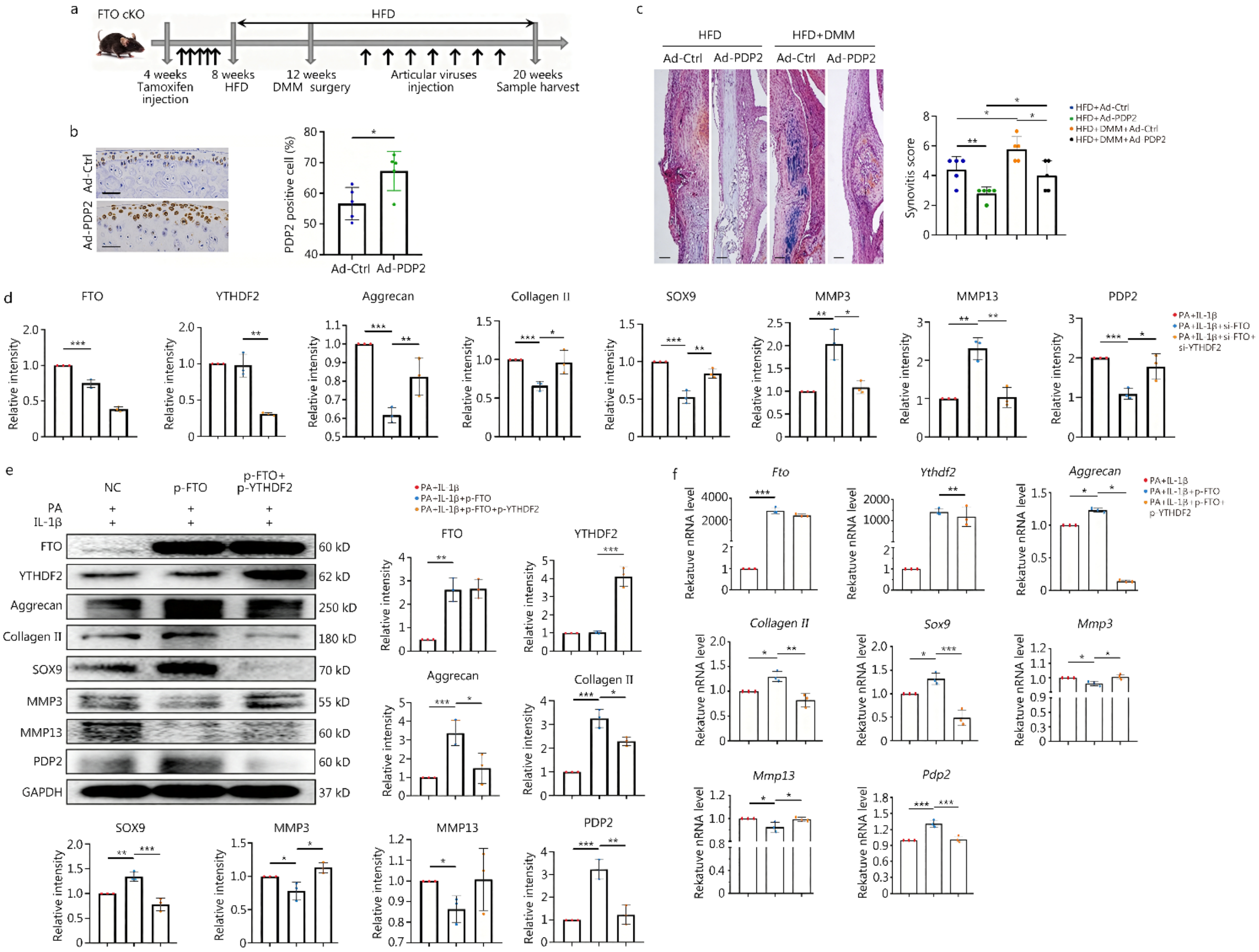

Fig. S7 Overexpression of PDP2 rescues cartilage degradation in HFD-fed and HFD+DMM FTO cKO mice, plus the role of YTHDF2 in OA. **a** Schematic illustration of HFD and HFD+DMM model design for FTO cKO mice. **b** IHC staining of PDP2 positive cells in Ad-Ctrl and Ad-PDP2 injected HFD, HFD+DMM mice (scale bar=200 μm). **c** Safranin O staining for synovium tissue and synovitis score of mice in Ad-Ctrl or Ad-PDP2-injected HFD, HFD+DMM mice (scale bar=200 μm). **d** Quantification of Western blotting results in **Fig. 8c**. **e** Representative Western blotting showing FTO, YTHDF2, Aggrecan, Collagen II, SOX9, MMP3, MMP13, and PDP2 expression after overexpression of YTHDF2 in chondrocytes treated with PA+IL-1β. **f** *Fto*, *Ythdf2*, *Aggrecan*, *Collagen II*, *Sox9*, *Mmp3*, *Mmp13*, and *Pdp2* mRNA levels after overexpression of YTHDF2 in chondrocytes treated with PA+IL-1β. The data are presented as mean±SEM. $^{*}P$<0.05, $^{**}P$<0.01, $^{***}P$<0.001. PDP2. Pyruvate dehydrogenase phosphatases 2; HFD. High fat diet; DMM. Destabilized medial meniscus; FTO. Fat mass and obesity associated gene; cKO. Conditional knockout; Ad-Ctrl. Adenovirus-control; Ad-PDP2. Adenovirus-PDP2 overexpression; YTHDF2. YTH N^6-methyladenosine RNA binding protein 2; PA. Palmitic acid; IL-1β. Interleukin-1β; si-FTO. Small interfering RNA-FTO; si-YTHDF2. Small interfering RNA-YTHDF2; P-FTO. Plasmid-FTO overexpression; p-YTHDF2. Plasmid-YTHDF2 overexpression; Aggrecan. Aggregating proteoglycan; Collagen II. Type II collagen; SOX9. Sex

determining region Y-box 9; MMP3. Matrix metalloproteinase 3; MMP13. Matrix metalloproteinase 13; GAPDH. Glyceraldehyde-3-phosphate dehydrogenase; IHC. Immunohistochemistry; SEM. Standard error of the mean

References

1. Ewels P, Magnusson M, Lundin S, Kaller M. MultiQC: summarize analysis results for multiple tools and samples in a single report. Bioinformatics. 2016;32(19):3047-8. https://doi.org/10.1093/bioinformatics/btw354.
2. Benjamini Y, Drai D, Elmer G, Kafkafi N, Golani I. Controlling the false discovery rate in behavior genetics research. Behav Brain Res. 2001;125(1-2):279-84. https://doi.org/10.1016/s0166-4328(01)00297-2.
